# Two-Dimensional Nanostructured Ti_3_C_2_T_x_ MXene for Ceramic Materials: Preparation and Applications

**DOI:** 10.3390/nano15030204

**Published:** 2025-01-27

**Authors:** Xiao-Tong Jia, Hong-Wei Xing, Xing-Wang Cheng, Zhao-Hui Zhang, Qiang Wang, Jin-Zhao Zhou, Yang-Yu He, Wen-Jun Li

**Affiliations:** 1 School of Materials Science and Engineering, Beijing Institute of Technology, Beijing 100081, China; 15732028671@163.com (X.-T.J.); 19833365013@163.com (H.-W.X.); 3120225558@bit.edu.cn (Q.W.); 18256194391@163.com (J.-Z.Z.); 3120215537@bit.edu.cn (Y.-Y.H.); lwj_material@163.com (W.-J.L.); 2 Tangshan Key Laboratory of High-Performance Metals and Ceramics, Tangshan Research Institute BIT, Tangshan 063000, China

**Keywords:** Ti_3_C_2_T_x_ MXene, synthesis, ceramic, mechanical property

## Abstract

Ti_3_C_2_T_x_ MXene, a novel two-dimensional transition metal carbide with nanoscale dimensions, has attracted significant attention due to its exceptional structural and performance characteristics. This review comprehensively examines various preparation methods for Ti_3_C_2_T_x_ MXene, including acid etching, acid–salt composite etching, alkali etching, and molten salt etching. It further discusses several strategies for interlayer exfoliation, highlighting the advantages and limitations of each method. The effects of these techniques on the nanostructure, surface functional groups, interlayer spacing, and overall performance of Ti_3_C_2_T_x_ MXene are evaluated. Additionally, this paper explores the diverse applications of Ti_3_C_2_T_x_ MXene in ceramic materials, particularly its role in enhancing mechanical properties, electrical and thermal conductivity, as well as oxidation and corrosion resistance. The primary objective of the review is to provide scientific insights and theoretical guidance for the preparation of Ti_3_C_2_T_x_ MXene and its further research and innovative applications in ceramic materials, advancing the development of high-performance, multifunctional ceramics.

## 1. Introduction

With the continuous advancement of materials science, two-dimensional (2D) materials have emerged as highly promising candidates for a wide range of applications in fields such as energy storage, sensors, catalysis, and biomedicine, owing to their unique structures and remarkable properties [1,2,3]. Among these, 2D transition metal carbides, nitrides, or carbonitrides, collectively known as MXene, have garnered significant attention due to their excellent electrical conductivity, thermal stability, mechanical properties, and the ability to modulate surface functionality [4,5]. Since the discovery of MXene in 2011, over 30 distinct compounds have been synthesized, including Ti_3_C_2_, Nb_2_C, V_4_C_3_, and Ti_3_N_4_. The general chemical formula for MXene is M_n+_1X_n_T_x_, where M represents a transition metal, X denotes carbon or nitrogen, A is typically an element from group III or IV, and T represents various surface functional groups. By selectively etching the A-layer elements from the MAX phase, MXene exhibits a unique, layered structure while displaying diverse physicochemical properties.

Among various MXene materials, Ti_3_C_2_T_x_ was the first to be discovered. Its unique structure and excellent performance have made it a focal point of research. Ti_3_C_2_T_x_ has a layered structure, consisting of alternating layers of Ti and C atoms. This structure not only preserves the high strength and superior mechanical properties of the MAX phase but also imparts notable conductivity, increased interlayer spacing, and a higher specific surface area [6,7]. These outstanding characteristics make Ti_3_C_2_T_x_ highly promising in the fields of electronics, thermodynamics, and mechanics. Moreover, Ti_3_C_2_T_x_ MXene offers flexible surface tunability, enabling the introduction of various functional groups such as -F, -OH, and -O. These groups provide it with high surface reactivity and adjustable physicochemical properties. The diversity in surface functionalization further enhances its compatibility with different matrix materials, expanding its potential for use in composite materials [8,9,10]. As one of the most extensively studied materials in the MXene family, Ti_3_C_2_T_x_ MXene has attracted significant attention, particularly regarding its preparation methods and applications in composite materials.

The synthesis process of Ti_3_C_2_T_x_ MXene plays a crucial role in determining its structure and performance. Therefore, developing efficient fabrication methods to produce Ti_3_C_2_T_x_ MXene with optimal properties has become a key focus of current research. Traditional synthesis methods typically rely on hydrofluoric acid (HF) etching, which effectively removes the A-layer elements from MAX phases, yielding high-quality Ti_3_C_2_T_x_ MXene. However, HF etching has drawbacks, including strong corrosiveness, significant environmental pollution, and low etching efficiency [11,12]. Consequently, in recent years, alternative etching methods have been proposed, such as acid–salt composite etching, alkaline etching, and molten salt etching, to improve the preparation efficiency and quality of MXene while reducing environmental harm [13,14,15,16]. After etching, additional delamination processes are often required to achieve single-layer or few-layer MXene [17].

Currently, the applications of Ti_3_C_2_T_x_ MXene primarily focus on supercapacitors, batteries, sensors, and electromagnetic shielding [18,19,20,21]. For example, Wan et al. [22] synthesized GaOOH/Ti_3_C_2_T_x_ composites via a hydrothermal method, using gallium nitrate (Ga(NO_3_)_3_) as the gallium source. As a supercapacitor electrode material, the GaOOH/Ti_3_C_2_T_x_ composite prepared with a 0.06 M gallium source concentration achieved a specific capacitance of 542.1 F·g^−1^. After 5000 charge–discharge cycles, the capacitance decreased by only 3.4%. Dai et al. [23] synthesized CoFe_2_O_4_/Ti_3_C_2_T_x_ MXene/CNFs composites by combining Ti_3_C_2_T_x_ with porous CoFe_2_O_4_ derived from metal–organic frameworks (MOFs) and integrating it with carbon nanofibers (CNFs). This composite effectively mitigated the volume expansion issue during battery charge–discharge cycles. It exhibited excellent rate performance, maintaining 332 mAh·g^−1^ at 20 A·g^−1^. After 1000 cycles, it retained a remarkable capacity of 972 mAh·g^−1^ at 2 A·g^−1^. Zhang et al. [24] synthesized 2D a-Fe_2_O_3_/Ti_3_C_2_T_x_ MXene composites through solvothermal and annealing methods. In this process, a-Fe_2_O_3_ was in situ grown on both the surface and interlayers of Ti_3_C_2_T_x_ MXene. The composite exhibited exceptional gas sensitivity (20.27 at 100 ppm) and ultra-low detection limits for H_2_S gas (10.19 ppb) at room temperature (25 °C). Although the application of Ti_3_C_2_T_x_ MXene in ceramic materials is still in its early stages, its unique 2D structure, superior mechanical properties, outstanding electrochemical performance, and tunable surface functionality render it highly promising for ceramic applications. As a reinforcing phase in ceramic materials, Ti_3_C_2_T_x_ MXene can significantly improve the strength and toughness of ceramics, while also imparting excellent oxidation and corrosion resistance. However, Ti_3_C_2_T_x_ MXene faces challenges in practical applications, particularly concerning thermal stability. Existing studies show that Ti_3_C_2_T_x_ MXene is prone to oxidation and degradation in high-temperature environments, limiting its use in ceramics, especially for applications requiring high-temperature sintering or long-term high-temperature stability. Specifically, under high-temperature conditions, surface oxidation of Ti_3_C_2_T_x_ MXene can lead to the destruction of its layered structure, weakening its adhesion to the ceramic matrix and reducing the overall material performance. Therefore, maintaining the integrity and functionality of Ti_3_C_2_T_x_ MXene during ceramic material fabrication is essential not only for ensuring its reinforcing effect in ceramics but also for enhancing the reliability and service life of ceramic-based composites in high-temperature environments. Finding ways to improve the thermal stability and high-temperature resistance of Ti_3_C_2_T_x_ MXene in ceramic material preparation is a critical challenge to address.

This review focuses on the preparation methods of Ti_3_C_2_T_x_ MXene and its applications in ceramic materials. Firstly, it explores various etching techniques, including acid etching, acid–salt composite etching, alkaline etching, and molten salt etching, while analyzing their working principles and effects on the structure and performance of MXene. Additionally, several emerging etching methods are introduced. Next, the review examines the delamination strategies for Ti_3_C_2_T_x_ MXene, with a focus on how different treatment methods effectively achieve its delamination. Finally, the paper summarizes the applications of Ti_3_C_2_T_x_ MXene in ceramic materials, particularly its potential to enhance the mechanical properties of ceramics. Through this review, we aim to provide scientific insights for further research and applications of Ti_3_C_2_T_x_ MXene, to lay the groundwork for its innovative applications in ceramics.

## 2. Etching Methods for Ti_3_C_2_T_x_ MXene

### 2.1. Acid Etching

**Figure 1:**
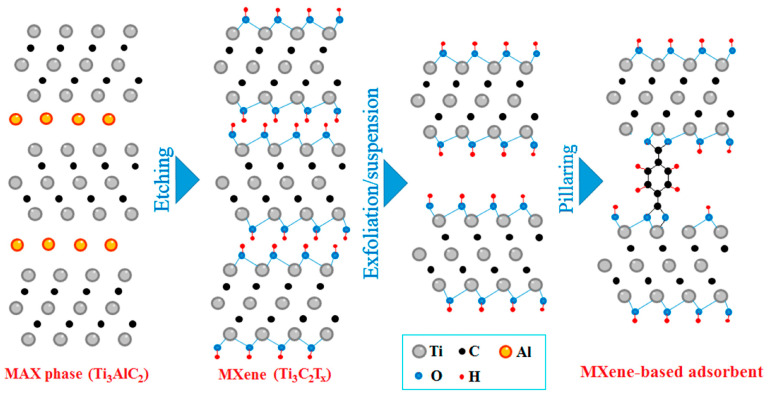
Schematic diagram of etching process [26].

In 2011, Naguib et al. [25] pioneered the use of hydrofluoric acid (HF) to etch Ti_3_AlC_2_, a member of the MAX phase family. Ti_3_AlC_2_ exhibits a typical M_n+1_AX_n_ structure, where M (Ti) elements are bonded by metallic bonds, X (C) elements are covalently bonded to the M (Ti) elements, and the A (Al) layer is connected to the M (Ti) layer by weaker metallic bonds. During the etching process, HF breaks the Ti–Al metallic bonds in Ti_3_AlC_2_, leading to the removal of the Al layer and the formation of 2D Ti_3_C_2_T_x_ MXene. The specific etching process is illustrated in Figure 1 [26]. The chemical reactions involved are as follows [27]:

Ti_3_AlC_2_ + 3HF → AlF_3_ + Ti_3_C_2_ + 2/3H_2_ (1)

Ti_3_C_2_ + 2H_2_O → Ti_3_C_2_(OH)_2_ + H_2_ (2)

Ti_3_C_2_ + 2HF → Ti_3_C_2_F_2_ + H_2_ (3)

**Figure 2:**
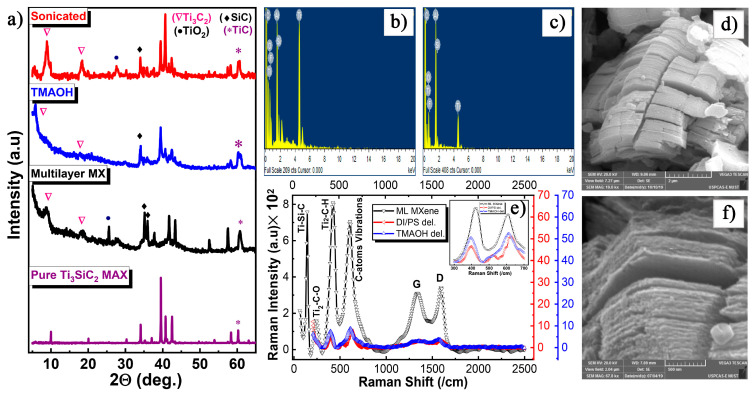
(**a**) XRD patterns of Ti_3_SiC_2_ MAX and Ti_3_C_2_T_x_ MXene; (**b**,**c**) EDX and (**e**) Raman spectra of MXene; (**d**,**f**) Microstructure of Ti_3_SiC_2_ after treatment with HF/H_2_O_2_ [28].

The Al-based MAX phase, Ti_3_AlC_2_, is the most commonly used precursor for etching Ti_3_C_2_T_x_ MXene. However, Si-based MAX phases, such as Ti_3_SiC_2_, can also be used to synthesize Ti_3_C_2_T_x_ MXene, although their application is less common due to the stronger Ti–Si bonds. In 2020, Noor et al. [28] successfully etched Ti_3_SiC_2_ to obtain Ti_3_C_2_T_x_ MXene using a mixed solution of HF and hydrogen peroxide (H_2_O_2_). In their study, single-layer MXene was obtained by ultrasonic cooling in an ice bath, without the need for intercalation agents. X-ray diffraction (XRD) analysis (Figure 2a) showed that the peak at 2θ = 39°, corresponding to the Ti_3_SiC_2_ MAX phase, disappeared, indicating the successful removal of the silicon layer and the formation of Ti_3_C_2_T_x_ MXene. Furthermore, the (002) peak of the delaminated Ti_3_C_2_T_x_ MXene shifted from 8.9° to 5.9°, with the interlayer spacing increasing from 9.87 Å to 15.22 Å, further confirming the delamination process. Energy-dispersive X-ray spectroscopy (EDX) in Figure 2b,c demonstrated a reduction in silicon content following the HF/H_2_O_2_ treatment. Raman spectroscopy (Figure 2e) revealed that after the removal of the silicon layer, the single-layer Ti_3_C_2_T_x_ MXene surface adsorbed terminal groups such as -O, -OH, and -F. These surface modifications increased the number of atoms within the unit cell and influenced the lattice structure of Ti_3_C_2_T_x_ MXene. The morphology of the etched Ti_3_C_2_T_x_ MXene, shown in Figure 2d,f, exhibited an accordion-like structure, similar to that obtained from etching Al-based MAX phases.

In 2022, Kiran et al. [29] introduced a novel electric-field-assisted flash sintering technique to rapidly synthesize the Ti_3_SiC_2_ MAX phase. They then used HF to etch the Ti_3_SiC_2_ MAX phase to obtain Ti_3_C_2_T_x_ MXene. The etching process began with HF treatment to remove surface oxides from the MAX phase, followed by high-energy ultrasonic treatment of the pre-treated solution using a high-power ultrasonic cell disruptor. Assisted by high-energy ultrasound, HF acid effectively disrupted the Ti–Si bonds in the MAX phase, resulting in the formation of layered Ti_3_C_2_T_x_ MXene with an interlayer spacing of 14.56 Å.

**Figure 3:**
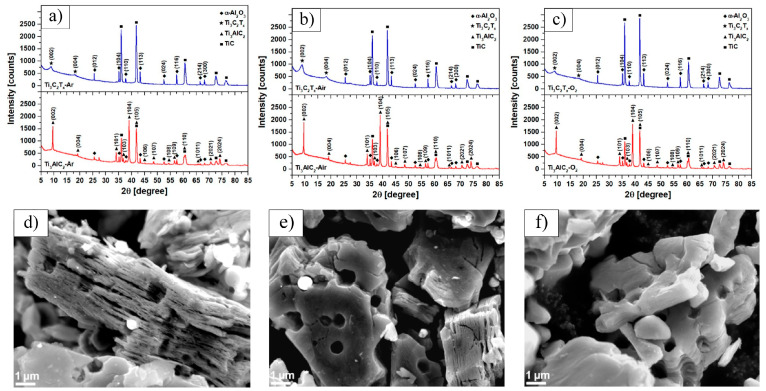
XRD patterns of Ti_3_AlC_2_/Ti_3_C_2_T_x_ obtained by treatment in: (**a**) Ar; (**b**) air; and (**c**) O_2_ environments; (**d**–**f**) SEM images of purified MXene particles [30].

A substantial body of research indicates that the quality of the precursor material plays a crucial role in determining the properties of Ti_3_C_2_T_x_ MXene after etching. Scheibe et al. [30] used a TiC–Ti_2_AlC powder mixture as the starting material and prepared three different Ti_3_AlC_2_ MAX phase precursors under argon, air, and oxygen atmospheres, named Ti_3_AlC_2_–Ar, Ti_3_AlC_2_–Air, and Ti_3_AlC_2_–O_2_, respectively. These precursors were then etched with HF to investigate how the structure of the MAX phase, influenced by different oxygen concentrations, affects the resulting Ti_3_C_2_T_x_ MXene. XRD (Figure 3a–c) and scanning electron microscopy (SEM) (Figure 3d–f) analyses revealed that the oxygen concentration significantly impacted the formation of α-Al_2_O_3_ inclusions between the MAX phase layers. As the oxygen concentration increased, the amount of a-Al_2_O_3_ inclusions also increased. During subsequent grinding and MXene preparation, these α-Al_2_O_3_ impurities were partially released, resulting in the formation of a porous structure in the MXene. However, some α-Al_2_O_3_ particles remained within the MXene and showed a tendency to grow, which reduced the specific surface area of the MXene. The specific surface areas of Ti_3_C_2_T_x_–Ar, Ti_3_C_2_T_x_–Air, and Ti_3_C_2_T_x_–O_2_ were measured as 13.7, 13.64, and 5.96 m²/g, respectively.

Wang et al. [31] demonstrated that Ti_3_AlC_2_ typically contains trace amounts of TiC, which can influence the lateral area and interlayer spacing of Ti_3_C_2_T_x_ after etching. These factors affect the mobility of charge carriers and the formation of a conductive network in Ti_3_C_2_T_x_, ultimately impacting its electromagnetic absorption properties. Therefore, controlling the TiC content in the Ti_3_AlC_2_ MAX to adjust the structure and properties of the resulting MXene represents a feasible strategy for tailoring its performance. In addition, Ahmadian et al. [32] investigated the effects of three different ball-milling parameters (milling time, interval time, and rotation speed) on the synthesis of Ti_3_AlC_2_ MAX phase and the resulting Ti_3_C_2_T_x_ MXene. The study revealed that the intermediate Ti and Al compounds generated during the milling process are critical for the formation of Ti_3_AlC_2_. If these intermediate compounds do not form, the subsequent formation of the MAX phase during the spark plasma sintering (SPS) process is hindered. Furthermore, the temperature increase during milling plays a critical role in facilitating the formation of Ti and Al compounds. The optimal ball milling process involved shorter rest periods. HF etching of Ti_3_AlC_2_ MAX phase, synthesized under different milling parameters, resulted in Ti_3_AlC_2_ MXene particles with sizes ranging from 0.1 μm to 0.5 μm. Experimental results indicated that the highest quality MAX and MXene were achieved using a milling speed of 400 rpm, a milling time of 18 h, and a 5 min interval time.

Additionally, the structure and properties of Ti_3_C_2_T_x_ MXene are significantly influenced by the etching time. Benchakara et al. [33] demonstrated that high-concentration HF etching induces expansion of the Ti_3_C_2_T_x_ MXene due to the generation of H_2_, resulting in a characteristic accordion-like structure. In contrast, low-concentration HF etching leads to insufficient etching, causing a more tightly stacked structure of Ti_3_C_2_T_x_ MXene. Furthermore, HF concentration plays a crucial role in regulating the content and distribution of surface functional groups on MXene, which in turn affects its surface properties. A higher HF concentration increases the -F content while decreasing the -O content in Ti_3_C_2_T_x_, thereby raising the overpotential for the hydrogen evolution reaction (HER). Density functional theory (DFT) calculations further indicate that as the ratio of fluorine substituting oxygen on the MXene surface increases, the free energy change of Ti_3_C_2_T_x_ increases monotonically, which reduces its HER activity and simultaneously diminishes its oxidation stability. Specifically, the introduction of -F functional groups reduces the surface energy of Ti_3_C_2_T_x_, decreasing its hydrophilicity and causing uneven electron cloud distribution, which in turn impacts its electronic conductivity and chemical stability. This alteration can lead to instability in the electrochemical performance of Ti_3_C_2_T_x_ under certain conditions.

**Figure 4:**
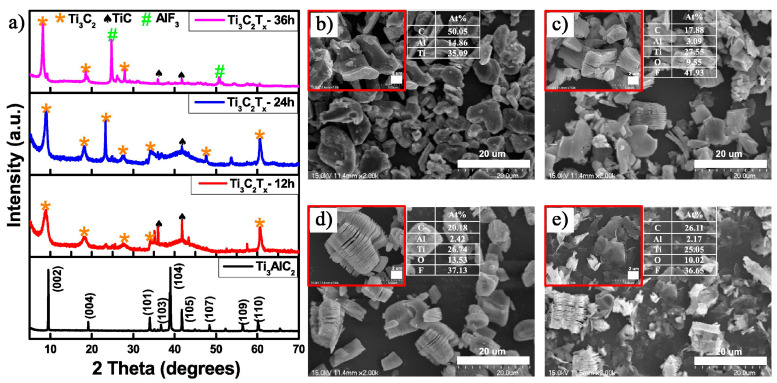
(**a**) XRD patterns of Ti_3_AlC_2_ and Ti_3_C_2_T_x_; SEM images of (**b**) Ti_3_AlC_2_ and (**c**–**e**) Ti_3_C_2_T_x_ prepared by HF etching for (**c**) 12 h, (**d**) 24 h, and (**e**) 36 h. The insets show lightly magnified SEM images [34].

Tran et al. [34] employed 49% HF to etch the Ti_3_AlC_2_ MAX phase at 5 °C and synthesize Ti_3_C_2_T_x_ MXene, exploring the influence of varying etching times on the quality of the resulting MXene. Figure 4 presents the XRD patterns and SEM images of the original Ti_3_AlC_2_ powder and the MXene prepared after various etching durations. From the intensity of the main peaks and the appearance of secondary peaks, it was found that Ti_3_C_2_T_x_ prepared after 24 h of etching exhibited superior quality compared to the other samples, a conclusion consistent with the SEM observations. Notably, after 12 h of etching, a clear TiC phase appeared as a secondary phase. After 36 h of etching, several characteristic peaks of Ti_3_C_2_T_x_ weakened, indicating structure degradation, which was also reflected in the SEM images. The Ti_3_C_2_T_x_ prepared displayed exceptional adsorption capabilities, achieving a removal efficiency of 91.9% within 5 min when 40 mg of Ti_3_C_2_T_x_ was added to a 20 μM methylene blue (MB) solution. This remarkable performance is closely associated with the presence of -F functional groups on the Ti_3_C_2_T_x_ surface. The -F groups confer a negative charge to the MXene surface, while the positively charged MB molecules interact electrostatically, facilitating the efficient removal of MB. This phenomenon highlights the critical role of surface functional groups in determining surface charge distribution and adsorption properties, with fluorine groups enhancing the adsorption capacity of MXene through polar interactions and electrostatic adsorption.

**Figure 5:**
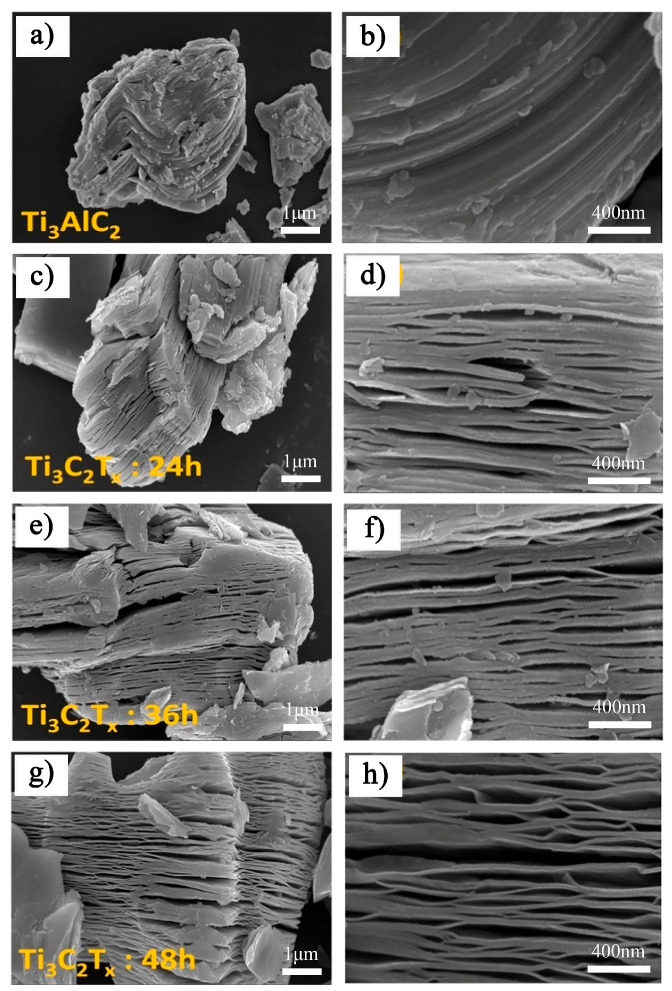
SEM images of (**a**,**b**) Ti_3_AlC_2_ MAX phase and (**c**–**h**) Ti_3_C_2_T_x_ etched for (**c**,**d**) 24 h, (**e**,**f**) 36 h, and (**g**,**h**) 48 h [35].

Similarly, Sumbe et al. [35] used 48% HF to etch Ti_3_AlC_2_ and examined the effect of etching time on the morphology of the resulting MXene. SEM images of Ti_3_AlC_2_ and Ti_3_C_2_T_x_ MXene prepared with varying etching times are shown in Figure 5. The results show that the MAX phase initially exhibits a tightly stacked layered structure. After 24 h of HF etching, some layers began to delaminate (Figure 5c,d), which was attributed to the selective removal of the aluminum layer. As the etching time increased to 36 h, more layers underwent delamination (Figure 5e,f). After 48 h of etching, over 90% of the layers had delaminated (Figure 5g,h). These findings suggest that the etching time should be carefully optimized, considering the structure of the original MAX phase, the concentration of the etching agent, and the desired properties of the MXene.

In addition to HF, researchers have explored alternative acidic solutions for the preparation of Ti_3_C_2_T_x_ MXene. In 2019, Scheibe et al. [36] investigated three distinct etching methods: (1) HF alone, (2) chlorosulfonic acid (ClHSO_3_), and (3) a combination of HF and chlorosulfonic acid to etch Ti_3_AlC_2_, yielding Ti_3_C_2_T_x_ MXene with varying surface functional groups. The study found that MXene etched with either HF or chlorosulfonic acid alone exhibited paramagnetic behavior. However, when a mixture of HF and chlorosulfonic acid was used, the MXene displayed two distinct types of free radicals and/or defects in their electron paramagnetic resonance spectra, as well as a mixed antiferromagnetic/paramagnetic behavior. This magnetic ordering was attributed to the sulfur-based functional groups (e.g., -S_2_ and -SO_3_H) introduced by the chlorosulfonic acid. The results highlighted a strong correlation between the surface functional groups and the properties of Ti_3_C_2_T_x_ MXene, particularly the significant alteration in its electronic structure and magnetic properties upon the introduction of sulfur-based groups. This modification opens new avenues for the potential use of Ti_3_C_2_T_x_ MXene in magnetism and electronics.

In 2011, Naguib et al. [25] conducted geometric optimization of hydroxylated and fluorinated Ti_3_C_2_T_x_ MXene layers using DFT. The results revealed that the monolayer Ti_3_C_2_ structure exhibited typical metallic behavior, whereas the hydroxylated and fluorinated Ti_3_C_2_T_x_ demonstrated semiconducting properties, with energy gaps of 0.05 eV and 0.1 eV between the valence and conduction bands, respectively. This suggests that by modulating the types of surface functional groups on Ti_3_C_2_T_x_, it is possible to effectively regulate its electronic cloud distribution and band gap, thereby tuning its performance. The -OH functional group enhances the hydrophilicity of MXene by forming hydrogen bonds with water molecules, thereby improving its performance in applications such as batteries and sensors. In contrast, the -F functional group significantly alters the electronic conductivity and stability of MXene, reducing its activity in oxidation reactions. Moreover, studies have shown that the presence of -F functional groups significantly lowers the surface energy, hydrophilicity, and electrical conductivity of Ti_3_C_2_T_x_ MXene, thus impacting its overall performance. Consequently, reducing the -F content on the surface of Ti_3_C_2_T_x_ MXene has become a key research focus [37]. In 2020, Xu et al. [38] successfully synthesized Ti_3_C_2_T_x_ MXene with -F functional groups on its surface by etching Ti_3_AlC_2_ with HF. The MXene was subsequently used as a co-catalyst for graphite-like carbon nitride (g-C_3_N_4_) and subjected to plasma treatment in a N_2_ and H_2_ atmosphere at 500 °C. This treatment effectively transformed the Ti–C functional groups on the Ti_3_C_2_T_x_ surface into Ti–O groups, particularly Ti^4+^, while removing some of the -F functional groups. This modification notably enhanced the photocatalytic activity of the Ti_3_C_2_T_x_ MXene, mainly due to Ti^4+^’s ability to capture photo-induced electrons from g-C_3_N_4_, improving the separation of electrons and holes under light irradiation, and ultimately enhancing photocatalytic performance. In the same year, Yang et al. [39] prepared Ti_3_C_2_T_x_ MXene from Ti_3_AlC_2_ via HF etching and further treated it with sodium hydroxide (NaOH) solution to form alkali Ti_3_C_2_T_x_. This alkali treatment converted some Ti_3_C_2_F_x_ into Ti_3_C_2_O_x_, effectively reducing the fluorination caused by the HF etching and significantly improving the performance of MXene in NH_3_ and humidity sensors. For the NH_3_ sensor, the response signal of the alkali-treated Ti_3_C_2_T_x_ to 100 ppm NH_3_ at 25 °C was twice that of the non-alkalized sample. This enhancement was primarily attributed to the adsorption of NH_3_ molecules on the Ti atoms of Ti_3_C_2_T_x_, where they form chemical bonds. Additionally, the presence of -O functional groups on the surface of alkali-treated Ti_3_C_2_T_x_ further strengthened the N–Ti bonding sites, increasing the adsorption capacity for NH_3_. In the humidity sensor, residual sodium ions (Na^+^) in the interlayer of the alkali-treated Ti_3_C_2_T_x_ reacted with water molecules to form [Na(H_2_O)m]^+^, promoting the adsorption of water molecules.

The acid etching method, particularly HF etching, remains one of the most widely used and extensively studied methods for preparing Ti_3_C_2_T_x_ MXene. This method offers the advantages of simplicity and operational convenience, effectively removing the Al or Si layers from the MAX phase to produce pure Ti_3_C_2_T_x_ MXene. Additionally, HF etching allows for the optimization of MXene’s interlayer spacing and surface functional groups by adjusting the etching conditions. However, the environmental and safety concerns associated with HF etching cannot be overlooked. HF solutions are highly corrosive and pose significant safety risks to personnel and the environment, especially in large-scale production where waste disposal becomes challenging. Moreover, the fluorine-rich functional groups produced by HF etching can reduce the chemical reactivity or electrochemical stability of MXene in certain applications. While HF etching remains a cornerstone of MXene research and applications, its environmental and safety drawbacks have driven researchers to explore more sustainable alternatives.

### 2.2. Acid–Salt Composite Etching

To overcome the drawbacks associated with directly using HF etching agents, researchers have explored alternative methods that generate HF in situ. In 2014, Ghidiu et al. [40] pioneered the use of a mixture of lithium fluoride (LiF) and hydrochloric acid (HCl) to generate HF in situ, successfully etching the Ti_3_AlC_2_ MAX phase and obtaining Ti_3_C_2_T_x_ MXene. The Ti_3_C_2_T_x_ produced via this method exhibited volumetric expansion upon hydration, allowing it to be molded like clay. After drying, it transformed into a highly conductive solid or could be rolled into thin films several tens of micrometers thick. These films exhibited a capacitance of up to 900 F/cm^3^ and demonstrated excellent cycling stability and rate performance. Compared to traditional HF etching methods, the LiF + HCl etching process facilitates the preparation of single-layer MXene sheets with large lateral dimensions. Studies showed that over 70% of the Ti_3_C_2_T_x_ MXene sheets had lateral dimensions ranging from 0.5 to 1.5 μm, with approximately 70% of the sheets consisting of 1 to 2 layers.

**Figure 6:**
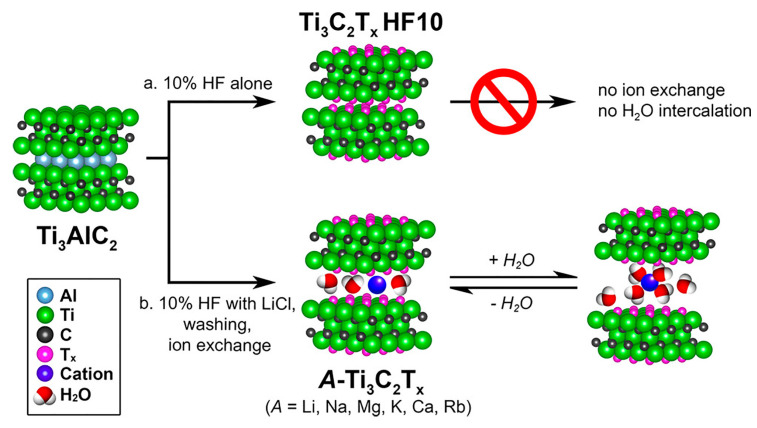
Different etchants used to obtain Ti_3_C_2_T_x_ [41].

In 2016, Ghidiu et al. [41] further investigated the impact of LiCl on the etching of Ti_3_AlC_2_ MAX phase into Ti_3_C_2_T_x_ MXene. Their study revealed the presence of Li^+^ ions between the MXene layers, which eliminated the need for subsequent intercalation steps and facilitated direct delamination. Additionally, as shown in Figure 6, they demonstrated successful ion exchange, replacing Li^+^ with cations such as K^+^, Na^+^, Rb^+^, Mg^2+^, and Ca^2+^. The study revealed that the intercalated cations induced changes in the material’s behavior, such as expansion at the unit lattice level in response to humidity, mimicking the swelling properties of clay minerals. In the same year, Sharma et al. [42] employed oxide molten liquid calorimetry to study the energy changes during the transformation of the MAX phase to MXene when etched with the HCl–LiF solution. Their findings showed that the MXene produced via this method exhibited exceptionally high stability. Compared to MXene etched with high-concentration HF, those treated with the HCl–LiF mixture displayed significantly larger interlayer spacing, which was primarily attributed to the intercalation of Li^+^ ions. Further work by Kumar et al. [43] in 2020 examined the effect of etching temperature (25 °C, 50 °C, and 80 °C) on the quality of Ti_3_C_2_T_x_ MXene synthesized from Ti_3_AlC_2_ using a concentrated hydrochloric acid (12 M) and LiF mixture solution. The study found that all MXene layers produced by etching had a thickness of less than 50 nm. As the etching temperature increased, both the etching of the MAX phase and the delamination of the MXene layers were enhanced. However, excessively high temperatures led to deterioration in the quality of the MXene, resulting in surface defects that reduced the active sites for pseudo-capacitive redox reactions and, consequently, a decrease in specific capacitance. Elevated temperatures also led to the formation of additional oxidized groups on the MXene surface, which hindered the formation of the electric double layer and further reduced specific capacitance. Electrochemical impedance spectroscopy analysis revealed that MXene etched at 50 °C exhibited superior performance in supercapacitors, with lower series resistance, smaller charge transfer resistance, and reduced Warburg impedance.

**Figure 7:**
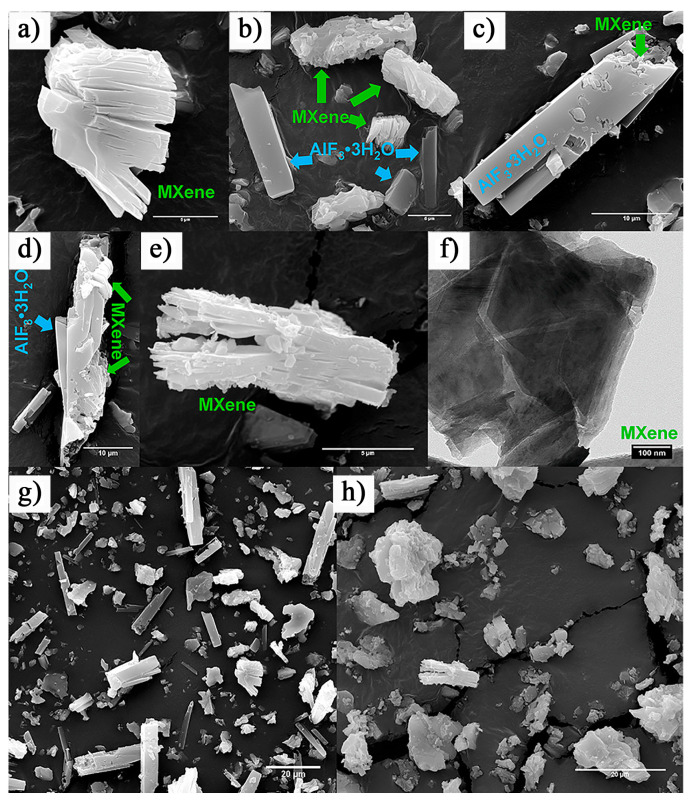
SEM images of (**a**–**d**,**g**) 5.0-CoF_2_/MAX-4 and (**e**,**h**) 5.0-CoF_3_/MAX-4; (**f**) Transmission electron microscope (TEM) of 3.6-CoF_2_/MAX-4 [44].

During the in situ etching process, where fluoride salts react with strong acids to generate HF, an undesirable byproduct that often forms is AlF_3_·3H_2_O. This byproduct can negatively affect the purity and properties of the Ti_3_C_2_T_x_ MXene. The typical accordion-like structure of pure Ti_3_C_2_T_x_ MXene is shown in Figure 7a, while Figure 7b–d illustrate the MXene structure containing AlF_3_·3H_2_O impurities [44]. In 2019, Cockreham et al. [44] proposed a method using cobalt fluorides (CoF_2_/CoF_3_) with HCl to in situ generate HF, successfully etching Ti_3_AlC_2_. They also investigated the factors influencing the formation of AlF_3_·3H_2_O impurities in the MXene, focusing on the etching environment. Their study revealed that the ionic strength of the etching solution is a critical factor driving the formation of AlF_3_·3H_2_O impurities. Specifically, the ionic strength directly impacts the thermodynamic stability of the AlF_3_ complex. When the ionic strength of the solution is between approximately 8.5 and 10 M, the AlF_3_ complex remains stable, promoting the formation of AlF_3_·3H_2_O impurities in the Ti_3_C_2_T_x_ MXene. Conversely, when the ionic strength is either below 8.5 M or above 10 M, the formation of AlF_3_·3H_2_O impurities is minimized, resulting in a product closer to pure MXene. This finding underscores the importance of carefully controlling the ionic strength during the etching process to minimize undesirable impurities and achieve higher-quality Ti_3_C_2_T_x_ MXene.

**Figure 8:**
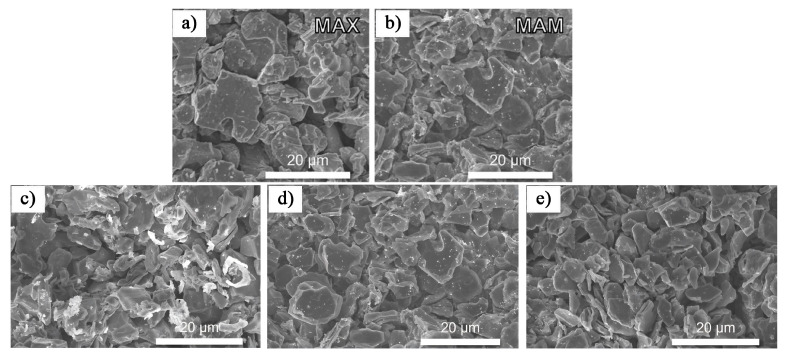
SEM images of: (**a**) MAX, (**b**) MAM, (**c**) MAM1, (**d**) MAM2, and (**e**) MAM3 [46].

In acid–salt composite etching methods, the choice of fluoride salts significantly influences the structure and properties of the resulting MXene. In 2020, Benchakar et al. [33] examined the effects of different fluoride salts, including LiF and iron fluoride (FeF_3_), on the surface chemistry of Ti_3_C_2_T_x_ MXene. The study demonstrated that etching with a LiF/HCl mixed solution produced conductive, clay-like MXene material, which facilitates further processing [45]. When LiF was replaced with FeF_3_, the conversion rate from MAX phase to MXene was accelerated, primarily due to the oxidative properties of Fe^3+^, which is beneficial for reducing the production costs of MXene. This occurs because, during Ti_3_C_2_T_x_ synthesis, Fe^3+^ can oxidize the surface Ti atoms, forming Ti_3_C_2_T_x_@TiO_2_-xF_2x_ composites. This oxidation process aids in the exfoliation of MXene, thus reducing the production cost. However, in contrast to the LiF/HCl method, the FeF_3_-based etching does not spontaneously exfoliate MXene in water. Furthermore, increasing the etching temperature and prolonging the etching time led to MXene dissolution, reducing the overall yield. In 2024, Yao et al. [46] employed high-intensity ultrasonic-assisted technology to successfully synthesize a series of MAX–MXene heterostructures (MAM) by adjusting the composition of etching agents (HCl and sodium fluoride (NaF)). Figure 8a,b show the microstructures of the original MAX phase and MAM. The original MAX phase (Figure 8a) displays an uneven size distribution and a compact, block-like structure. After etching with NaF+HCl, the MAX phase begins to exfoliate and break down, partially forming MAM nanostructures, as shown in the low-magnification SEM image in Figure 8b. Figure 8c–e show MAM products prepared with varying amounts of etching agents. It is evident that as the etching agent concentration increases, the MAM layer thickness decreases, and surface impurities, such as aluminum fluoride (AlF_3_) compounds containing O and C, are reduced [47]. Furthermore, the study found that the MXene generated on the MAM surface significantly enhanced electron transport efficiency, resulting in improved conductivity (with a minimum resistance of just 7 kΩ). The material’s surface also featured abundant adsorption sites, which greatly enhanced its gas sensing performance.

**Figure 9:**
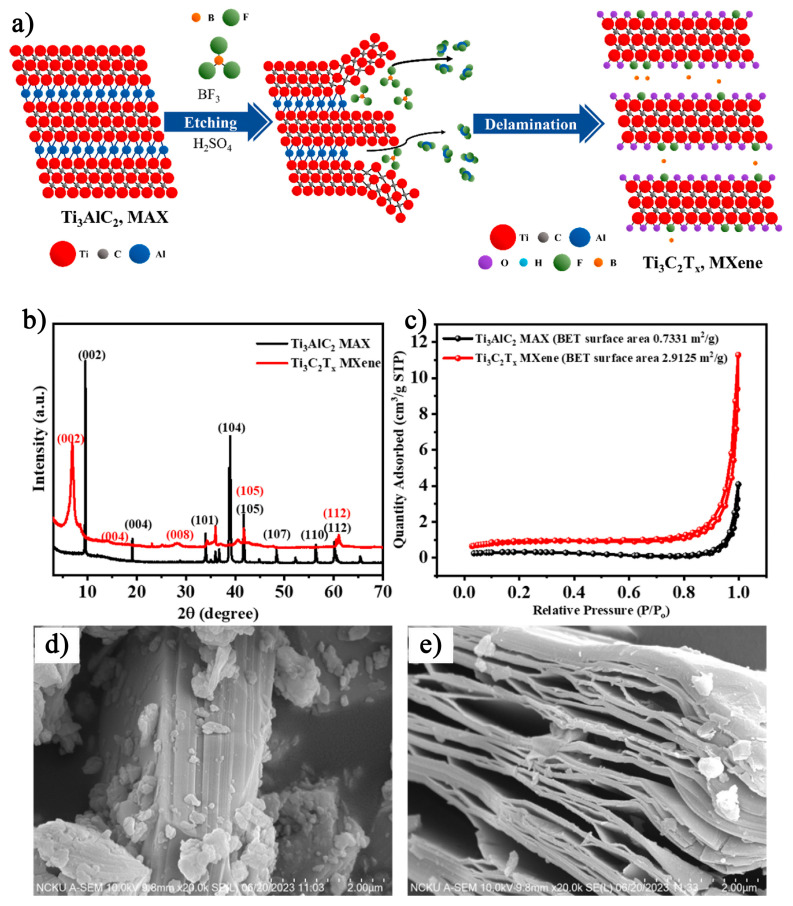
(**a**) Schematic diagram of etching process of B-doped Ti_3_C_2_T_x_ MXene; (**b**) XRD patterns and (**c**) BET of Ti_3_AlC_2_ MAX and B-doped Ti_3_C_2_T_x_ MXene; SEM images of (**d**) Ti_3_AlC_2_ MAX and (**e**) B-doped Ti_3_C_2_Tx MXene [48].

In recent years, researchers have expanded beyond traditional HCl etching methods to explore composite etchants that combine other acids with fluoride salts. In 2024, Huang et al. [48] pioneered the use of Lewis acids and boron trifluoride (BF_3_) as electron-deficient etchants in sulfuric acid (H_2_SO_4_) solution to etch aluminum from Ti_3_AlC_2_ MAX phase, resulting in the formation of B-doped Ti_3_C_2_T_x_ MXene. The specific etching process is shown in Figure 9a. As seen in the XRD results in Figure 9b, the incorporation of B led to broader diffraction peaks and an increase in the interlayer spacing of the material. Further analysis of the specific surface area of Ti_3_AlC_2_ MAX phase and Ti_3_C_2_T_x_ MXene (Figure 9c) revealed that after etching, the specific surface area of the B-doped Ti_3_C_2_T_x_ MXene (2.9125 m^2^/g) was approximately four times greater than that of the original MAX phase (0.7331 m^2^/g). This increase in surface area is attributed to the bonding of exposed Ti with elements from the etching solution, leading to functionalization with -F, -O, and -B groups, which in turn enhances the surface area. Additionally, a comparison of Figure 9d,e reveals that the etched Ti_3_C_2_T_x_ MXene transitioned from the dense, stacked structure of Ti_3_AlC_2_ to a more distinctly delaminated structure. The B-doped Ti_3_C_2_T_x_ MXene exhibited a larger interlayer spacing, with B inserted between the MXene layers. This enhancement contributed to a greater pseudocapacitive effect, improving the charge transfer and storage capabilities of the MXene electrode. At a current density of 1 A/g, the material demonstrated an energy density of 3.97 Wh/kg, a power density of 383.90 W/kg, and a maximum capacitance of 396 F/g. Additionally, the MXene showed excellent capacitance retention, highlighting its promising supercapacitor characteristics.

In 2024, Jee et al. [49] developed a more environmentally friendly method for synthesizing Ti_3_C_2_T_x_ MXene by employing a weak acid, phosphoric acid (H_3_PO_4_), in combination with LiF, zinc fluoride (ZnF_2_), and copper fluoride (CuF_2_) as etchants. The specific etching reaction pathway is as follows:

H_3_PO_4_ → 3H^+^ + PO4^3−^/CuF_2_ → Cu^2+^ +2F^−^ (4)

Al^3+^ + 3F^−^ → AlF_3_/Al^3+^ + PO4^3−^ → AlPO_4_ (5)

Ti_3_AlC_2_ → Al^3+^ +3e^−^ + Ti_3_C_2_/Cu^2+^ +2e^−^ → Cu (6)

In this method, Al in the Ti_3_AlC_2_ MAX phase is removed through two etching mechanisms. First, Al reacts with phosphate ions (PO_4_^3−^) and fluoride ion (F^−^) to form aluminum phosphate (AlPO_4_) and aluminum fluoride (AlF_3_). Second, lithium ions (Li^+^), zinc ions (Zn^2+^), and copper ions (Cu^2+^) facilitate the remove of Al from Ti_3_AlC_2_ via an electrochemical displacement reaction. The combination of H_3_PO_4_ and CuF_2_ demonstrates significantly higher Al removal efficiency compared to LiF and ZnF_2_. This enhanced efficiency is attributed to the superior ionization tendency of Cu^2+^. The H_3_PO_4_/CuF_2_ combination allows for rapid etching of Ti_3_AlC_2_ MAX at low temperatures (35 °C) within just 4 h, achieving substantial etching effects. In contrast, traditional HF etching methods typically require more than 24 h to achieve similar results.

Compared to traditional HF etching, the acid–salt composite etching method mitigates some of the limitations associated with HF etching. By introducing fluoride salts into an acidic solution, HF is generated in situ, thereby avoiding the direct use of HF and reducing both safety and environmental risks. This method also lowers the fluorine content on the MXene surface, improving its chemical stability and electrochemical performance. Furthermore, the presence of salts helps regulate ion concentration and diffusion rates in the reaction system, enhancing etching efficiency and ensuring more uniform product quality. However, despite these advantages, the method still operates within an acidic environment, meaning its environmental impact is not entirely mitigated. Additionally, in comparison to single-acid etching methods, the acid–salt composite etching approach introduces greater process complexity, requiring precise control over the acid-to-salt ratio and other reaction conditions. While this method addresses some of the shortcomings of traditional HF etching, further refinement is necessary to enhance its efficiency and sustainability.

### 2.3. Alkaline Etching

**Figure 10:**
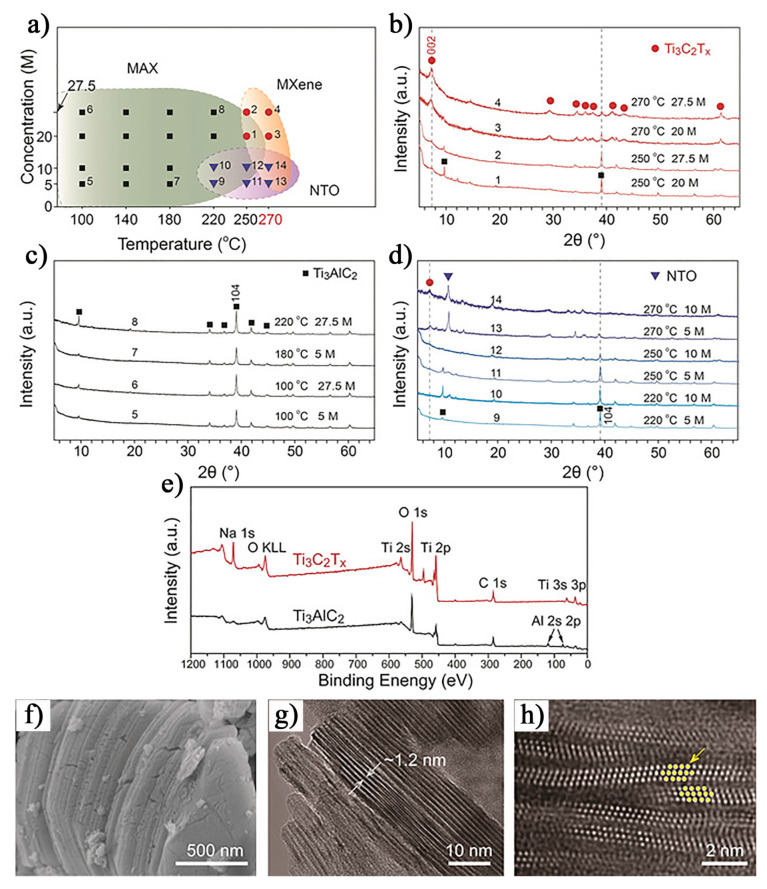
(**a**) Preparation conditions of NaOH–Ti_3_C_2_T_x_; XRD of (**b**) MXene, (**c**) MAX, and (**d**) NaOH–Ti_3_C_2_T_x_, (**e**) XPS of Ti_3_AlC_2_ and Ti_3_C_2_T_x_; (**f**–**h**) SEM images of the NaOH–Ti_3_C_2_T_x_. The bright spot in figure (**h**) represents the position of Ti [50].

Compared to traditional MAX phase etching methods, it is theoretically possible to etch the A-layer using an alkaline solution. In 2018, Li et al. [50] successfully etched MAX phases using a NaOH-assisted hydrothermal process, resulting in Ti_3_C_2_T_x_ MXene with a purity of up to 92% (NaOH–Ti_3_C_2_T_x_). Since this etching process does not involve fluoride compounds, the resulting MXene surface functional groups are primarily -OH and -O, rather than -F. The hydroxyl and oxygen functional groups contribute to the formation of pseudo-capacitive effects associated with hydrogen ion (H^+^), thereby enhancing the capacitive performance of the MXene. Testing results indicate that in 1 M H_2_SO_4_, the volumetric capacitance of NaOH–Ti_3_C_2_T_x_ (511 F/cm^3^, 1.63 mV/s) is 43.9% higher than that of Ti_3_C_2_T_x_ (355 F/cm^3^, 2.2 g/cm^3^). Additionally, after 10,000 cycles, it retains 89.1% of its capacitance, demonstrating excellent stability and durability. However, it is important to note that the formation of aluminum hydroxide and hydrated alumina is inevitable in the alkaline etching environment, which may hinder MXene synthesis. Despite this, under high-temperature and high-pressure conditions, concentrated alkaline solutions can effectively dissolve aluminum oxides and hydroxides, thus promoting MXene synthesis. As shown in Figure 10a–d, the content of Ti_3_C_2_T_x_ increases with rising temperature. This can be attributed to the endothermic nature of the dissolution reactions of Al oxides and hydroxides, where higher temperatures facilitate these reactions, thereby promoting MXene synthesis [51,52]. In contrast, lowering the temperature halts the reaction, indicating that temperature is a critical factor influencing the progress of the etching process. Additionally, at a constant reaction temperature, decreasing the alkaline concentration leads to a significant increase in the water content of the obtained MXene samples. Further experimental results reveal that when Ti_3_C_2_T_x_ is treated with 27.5 M NaOH at 270 °C, the {002} interlayer spacing increases to 24 Å, which is considerably larger than the {002} interlayer spacing of Ti_3_C_2_T_x_ prepared by the HF method (20 Å), as reported by Naguib et al. [25] and Mashtalir et al. [53]. This suggests that sodium ion (Na^+^) from the high-concentration NaOH solution is incorporated into the interlayer structure of the MXene. This hypothesis was supported by X-ray photoelectron spectroscopy (XPS) analysis (Figure 10e), where a decrease in the Al signal of Ti_3_C_2_T_x_ was accompanied by the appearance of a Na signal. SEM images in Figure 10f–h show that the NaOH–Ti_3_C_2_T_x_ exhibits a closely stacked layered structure, distinct from the typical accordion-like structure obtained using high-concentration (50%) HF etching, and more similar to the MXene structure formed by the slow H_2_ generation during low-concentration (5%) HF treatment. The resulting Ti_3_C_2_T_x_ has lateral dimensions of approximately 80–200 nm, a specific surface area of 16 m^2^/g (higher than the 9 m^2^/g of the original Ti_3_AlC_2_), and an interlayer spacing of approximately 1.6 nm (larger than the 0.93 nm of the original Ti_3_AlC_2_). These results suggest that the NaOH-assisted hydrothermal method can effectively tune the structure and performance of Ti_3_C_2_T_x_, providing new insights for MXene synthesis and application.

**Figure 11:**
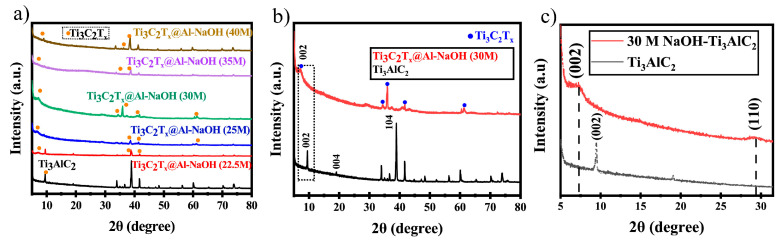
XRD patterns of (**a**) Ti_3_AlC_2_ and Ti_3_C_2_T_x_@Al-NaOH MXenes and (**b**,**c**) MAX phase and Ti_3_C_2_T_x_@Al-NaOH (30 M) MXene [54].

In 2024, Khan et al. [54] successfully synthesized Ti_3_C_2_T_x_@Al–NaOH composites using an alkaline-assisted hydrothermal method at various NaOH concentrations (22.5 M, 25 M, 30 M, 35 M, and 40 M) at 280 °C. As shown in the XRD patterns in Figure 11a–c, most of the diffraction peaks in the etched products disappeared compared to pure Ti_3_AlC_2_, and the peaks between 5° and 80° were significantly weakened and broadened. These results suggest that the reaction between Ti_3_AlC_2_ and NaOH at high temperature led to the formation of a new structure. Furthermore, the Ti_3_C_2_T_x_@Al–NaOH (30 M) sample exhibited a slight shift in the (002) peak position, along with the appearance of a characteristic (104) peak, further confirming the surface functionalization of the MXene with -OH and -O groups. The presence of these functional groups enhances the pseudocapacitive effect of Ti_3_C_2_T_x_. Further investigations revealed that the Ti_3_C_2_T_x_@Al–NaOH sample synthesized at a NaOH concentration of 30 M with 15 h of etching demonstrated a capacitance increase of approximately 465% compared to Ti_3_C_2_T_x_ MXene synthesized using the traditional HF etching method. This significant enhancement in capacitance indicates that Ti_3_C_2_T_x_@Al–NaOH samples have considerable potential for use in supercapacitors and other energy storage devices.

In 2024, Colkesen et al. [55] utilized both potassium hydroxide (KOH) alkaline solution and HF as etchants to etch Ti_3_AlC_2_, thereby obtaining Ti_3_C_2_T_x_ with different surface functional groups. The etching equations for both methods are as follows:

Ti_3_AlC_2_ + 3HF → Ti_3_C_2_T_x_ + 3/2H_2_ + AlF_3_ (7)

Ti_3_AlC_2_ + KOH + H_2_O → Ti_3_C_2_T_x_ + KAlO_2_ + 3/2H_2_ (8)

**Figure 12:**
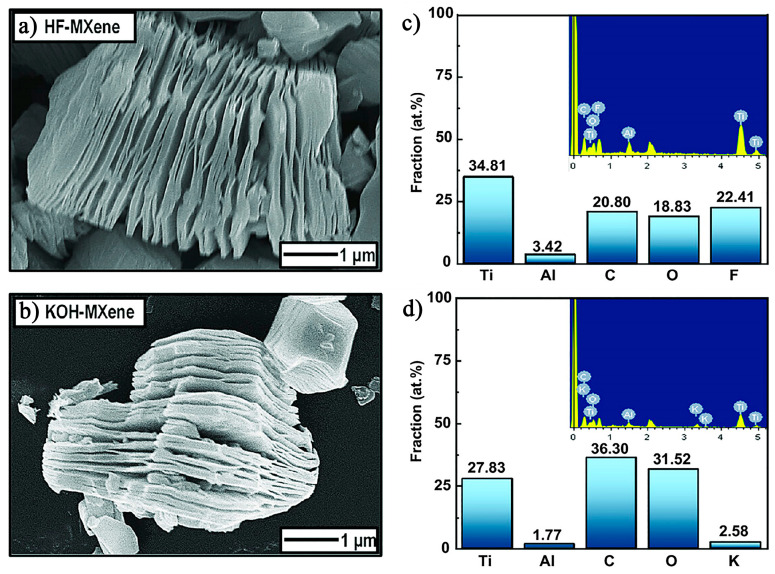
(**a**) SEM images and (**c**) EDS analysis of HF–MXene; (**b**) SEM images and (**d**) EDS analysis of KOH–MXene [55].

Figure 12a,c show the SEM images of Ti_3_C_2_T_x_ MXene synthesized by HF and KOH etching (i.e., HF–MXene and KOH–MXene). Both exhibit the characteristic accordion-like structure, confirming the successful synthesis of 2D MXene nanosheets. Figure 12b,d present the energy dispersive spectroscopy (EDS) results for HF–MXene and KOH–MXene. Compared to HF–MXene (3.42 at.%), the Al content in KOH–MXene (1.77 at.%) is significantly reduced, indicating that KOH is more effective than HF in removing metallic Al from the MAX phase. Additionally, the study reveals that the O content in MXene primarily originates from deionized water washing. The oxygen content in KOH–MXene (31.52 at.%) is notably higher than in HF–MXene (18.33 at.%). This can be attributed to the fact that during HF etching, the -F groups occupy part of the exposed Ti surface, leaving only the remaining free surface for -O groups. As a result, the oxygen content in HF–MXene is considerably lower than in KOH–MXene. Further investigation indicates a significant difference in the ability of HF–MXene and KOH–MXene to remove Sr^2+^. HF–MXene achieves a maximum Sr^2+^ removal rate of 82.0%, while KOH–MXene removes 99.3% of Sr^2+^. The Sr^2+^ adsorption capacity of HF–MXene is 180.48 mg/g, while KOH–MXene’s is 218.40 mg/g. These data suggest that KOH–MXene demonstrates superior adsorption capacity for Sr^2+^ compared to HF–MXene, which is closely related to the differences in surface functional groups. During the adsorption process, Sr^2+^ is effectively adsorbed onto the KOH–MXene surface via electrostatic interactions with -OH and -O functional groups. In contrast, the -F functional groups on the surface of HF–MXene reduce its ability to adsorb Sr^2+^. Additionally, KOH–MXene outperforms HF–MXene in terms of reusability. KOH–MXene retains over 97% removal efficiency after five consecutive adsorption cycles, whereas HF–MXene stabilizes around 80%. These results demonstrate that KOH–MXene has superior adsorption performance and recyclability, highlighting its potential for environmental pollution remediation.

Alkaline etching has emerged as a novel method for preparing MXene, using high-concentrated alkaline solutions to etch the MAX phase and produce MXene materials with fluoride-free terminations. This fluoride-free structure significantly enhances MXene’s hydrophilicity and environmental compatibility, broadening its potential applications in specialized fields such as biomedicine. Additionally, alkaline etching typically offers high reaction efficiency, enabling rapid etching within a relatively short time frame. However, several challenges remain. First, alkaline etching generally requires high concentrations of alkaline solutions, elevated temperatures, and extended processing times, leading to high energy consumption and increased safety risks in large-scale production. Second, MXene synthesized using alkaline etching often exhibits a characteristic accordion-like morphology, necessitating further intercalation and delamination steps to obtain single- or few-layer MXene. This adds to the complexity and costs of the process. Additionally, alkaline waste generated during etching can pose environmental risks if not properly managed. Future research should focus on reducing energy consumption and waste treatment costs while optimizing the etching process to enhance the quality and safety of MXene.

### 2.4. Molten Salt Etching

**Figure 13:**
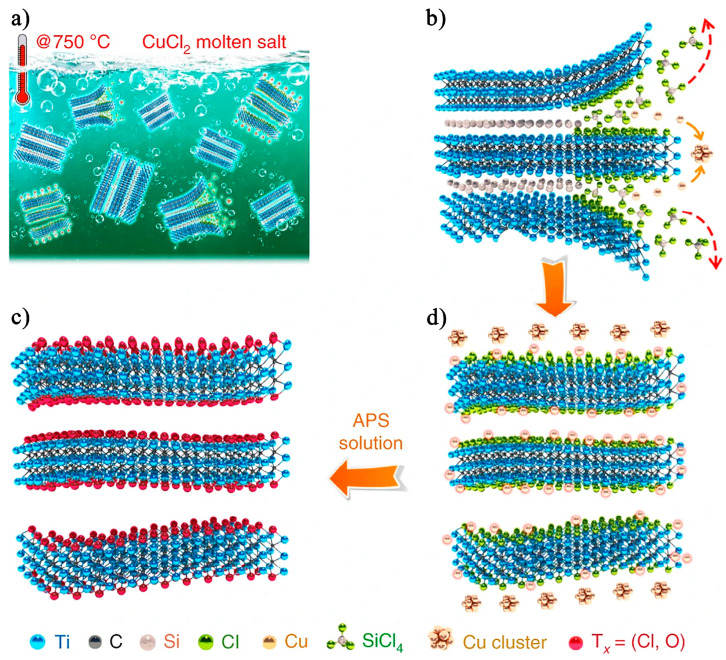
(**a**) Ti_3_SiC_2_ MAX phase is immersed in CuCl_2_ Lewis molten salt; (**b**,**c**) The reaction between Ti_3_SiC_2_ and CuCl_2_ results in the formation of Ti_3_C_2_T_x_ MXene; (**d**) MS-Ti_3_C_2_T_x_ MXene is obtained after further washing in ammonium persulfate (APS) solution [56].

In 2020, Li et al. [56] introduced a novel method for synthesizing MXene by controlling the redox reaction of A-site elements in MAX phases using Lewis acidic molten salts. This innovative method not only expanded the synthesis routes for MXene but also significantly improved its electrochemical performance, particularly in non-aqueous electrolytes. Furthermore, this method allows for the selective tuning of MXene’s surface functional groups by choosing specific Lewis acid etchants. The etching steps are illustrated in Figure 13, with the corresponding reaction given as follows:

Ti_3_SiC_2_ + 2CuCl_2_ → Ti_3_C_2_ + SiCl_4_↑ + 2Cu (9)

Ti_3_C_2_ + CuCl_2_ → Ti_3_C_2_Cl_2_ + Cu (10)

**Figure 14:**
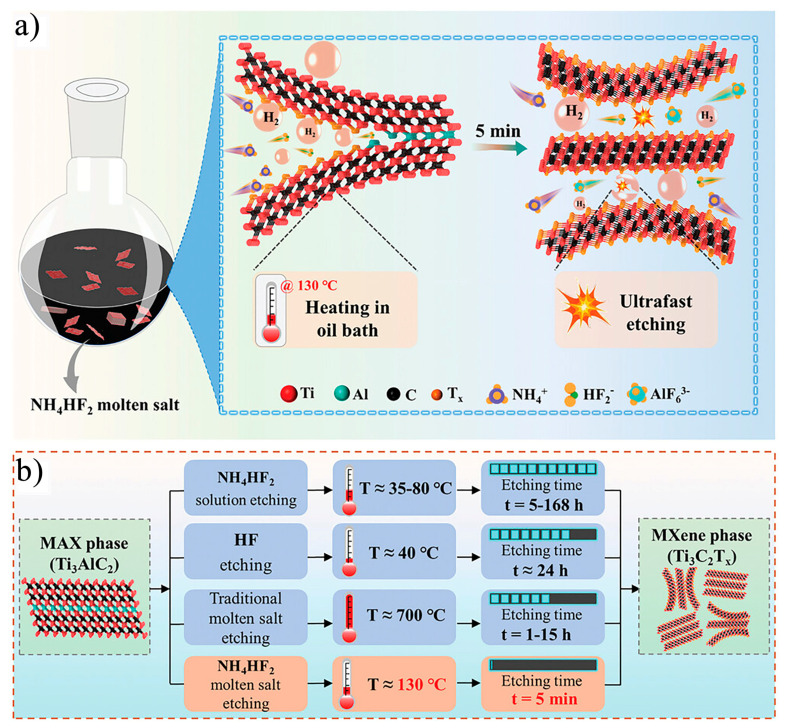
(**a**) Schematic illustration of LTMS etching strategy; (**b**) comparison between LTMS etching strategy and traditional etching method [57].

Traditional MXene synthesis methods typically require extended processing times and impose stringent etching conditions, resulting in low efficiency and limited scalability. These factors hinder the commercialization and broader applications of MXene. Therefore, there is a pressing need for more efficient methods that allow for the rapid synthesis of MXene. In 2024, Wang et al. [57] developed a rapid, large-scale synthesis method for various MXenes, including V_4_C_3_T_x_, Nb_4_C_3_T_x_, Mo_2_TiC_2_T_x_, Mo_2_CT_x_, and Ti_3_C_2_T_x_, using ammonium bifluoride (NH_4_HF_2_) as the etchant in combination with ultra-fast low-temperature molten salt (LTMS) etching. In a single reaction, a yield of Ti_3_C_2_T_x_ can exceed 100 g, with the resulting Ti_3_C_2_T_x_ exhibiting a multi-layer structure with an interlayer spacing of 1.27 nm. The etching process is depicted in Figure 14a, with the reaction equation as follows:

Ti_3_AlC_2_ + 3NH_4_HF_2_ → Ti_3_C_2_ + (NH_4_)_3_AlF_6_ + 3/2H_2_ (11)

Figure 14b highlights three key advantages of the LTMS method over conventional methods. First, molten NH_4_HF_2_ exhibits good fluidity, enabling it to quickly penetrate the Al atomic layers within the MAX phase. This accelerates the removal of by-products such as (NH_4_)_3_(AlF)_6_, preventing interlayer clogging in the MXene. Second, the H_2_ produced during the etching process increases the interlayer spacing of the MXene, which enhances the accessibility of Al atomic reaction sites, further promoting the etching reaction. Finally, the LTMS etching process is exothermic, and the heat generated during the reaction rapidly raises the temperature of the etching system, accelerating the etching kinetics and completing the reaction in just 5 min. In contrast, traditional high-temperature molten salt methods typically require temperatures above 700 °C, whereas the LTMS method achieves the etching of Ti_3_AlC_2_ at a relatively low temperature of 130 °C, offering a significant advantage in terms of energy efficiency. These characteristics make the LTMS method highly promising for cost-effective, large-scale industrial production of MXene.

Molten salt etching has gained attention as a novel, environmentally friendly method for MXene preparation. Unlike traditional acid or alkali etching, this method employs high-temperature molten salts to etch the MAX phase, eliminating the need for hazardous acid or alkaline solutions and significantly reducing risks to both personnel and the environment. The method also offers high etching efficiency and excellent process controllability, making it an attractive option for the large-scale, environmentally friendly production of MXene. However, molten salt etching has some limitations. The process requires high temperatures, leading to substantial energy consumption, and the selection and disposal of molten salts must be carefully managed to minimize environmental impacts. Despite its potential, further research is needed to address challenges related to energy efficiency, salt recycling, and waste management, paving the way for a more sustainable and cost-effective production process.

### 2.5. Other Etching Methods

**Figure 15:**
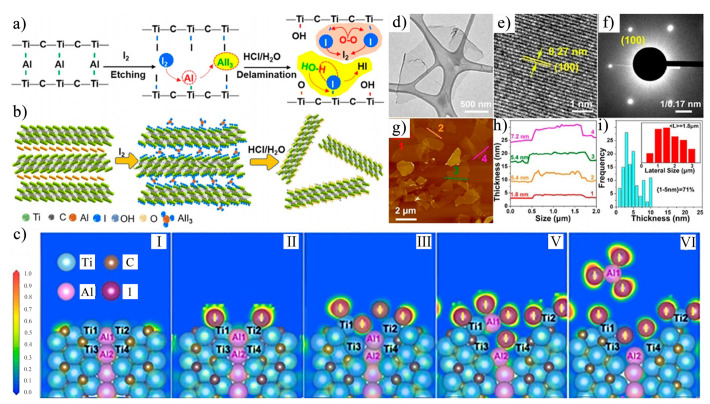
(**a**) Iodine etching process; (**b**) layered process; (**c**) mechanism of etching process; (**d**) TEM image; (**e**) HRTEM image; (**f**) selected area electron diffraction (SAED) pattern; (**g**) AFM image; (**h**) height profile; (**i**) thickness distribution [58].

In recent years, researchers have explored a variety of etching methods for synthesizing Ti_3_C_2_T_x_ MXene, proposing several novel techniques that differ significantly from traditional methods. In 2021, Shi et al. [58] introduced an innovative method for synthesizing Ti_3_C_2_T_x_ MXene using iodine, resulting in MXene with oxygen-rich terminal groups. The etching mechanism of this method is illustrated in Figure 15a–c, and the corresponding chemical reactions are as follows:

Ti_3_AlC_2_ + (x + 3)/2I_2_ → Ti_3_C_2_I_x_ + AlI_3_ (12)

Ti_3_C_2_I_x_ + x/2O_2_ → Ti_3_C_2_O_x_ + x/2I_2_ (13)

Ti_3_C_2_I_x_ + xH_2_O → Ti_3_C_2_(OH)_x_ + xHI (14)

AlI_3_ + 3H_2_O → Al(OH)_3_ + 3HI (15)

Al(OH)_3_ + 3HCl → AlCl_3_ + 3H_2_O (16)

Characterization of the iodine-etched Ti_3_C_2_T_x_ MXene (IE–MXene) was performed using TEM and AFM, with the results presented in Figure 15d–i. These characterizations revealed that the crystal structure of IE–MXene remained intact, with no obvious lattice defects, owing to the mild etching treatment in anhydrous solvents. Furthermore, as shown in Figure 15h,i, more than 71% of the layers in IE–MXene exhibited a thickness of less than 5 nm, with an average lateral size of 1.8 μm, significantly larger than the MXene produced by traditional HF etching (with a lateral size typically under 500 nm) [59,60]. The larger flake size and thinner structure of IE–MXene facilitate the preparation of MXene films through dispersion filtration. Compared to HF-etched MXene, IE–MXene exhibited superior environmental stability due to the absence of fluoride ions and the presence of oxygen-rich functional groups on its surface. These oxygen-containing groups not only enhanced the environmental stability of IE–MXene but also provided active sites, thereby facilitating the adsorption of H^+^ and significantly improving its specific capacitance.

**Figure 16:**
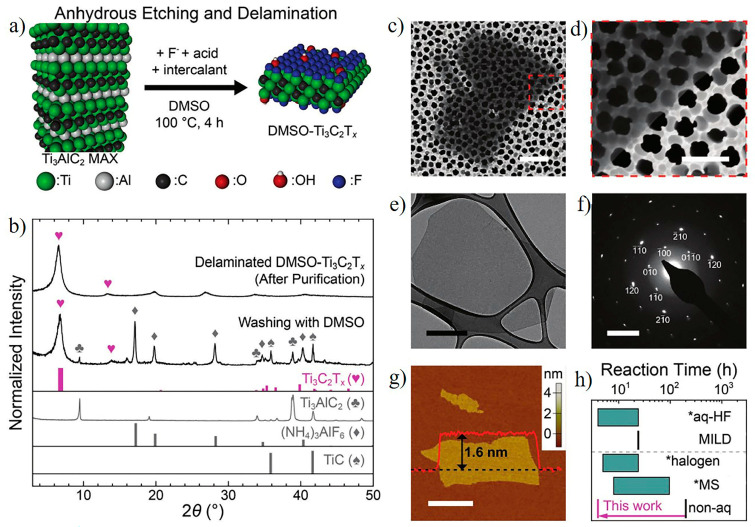
(**a**) Schematic diagram of DMSO etching process; (**b**) XRD patterns; (**c**,**d**) SEM, (**e**) TEM, (**f**) SAED, and (**g**) AFM images of DMSO–Ti_3_C_2_T_x_; (**h**) comparison of reaction times [61].

In 2022, Oh et al. [61] proposed a rapid and efficient method for synthesizing Ti_3_C_2_T_x_ MXene using anhydrous dimethyl sulfoxide (DMSO) solution. This method employs DMSO as a solvent, combined with ammonium bifluoride (NH_4_HF_2_) and methanesulfonic acid (CH_3_SO_3_H) as etchants, to achieve high-quality Ti_3_C_2_T_x_ etching at 100 °C within just 4 h. The resulting DMSO–Ti_3_C_2_T_x_ exhibits a flake size of 4.2 ± 2.2 µm, with the etching process detailed in Figure 16a. The XRD pattern in Figure 16b confirms the complete etching of Ti_3_AlC_2_. SEM images in Figure 16c,d reveal the typical morphology of exfoliated single-layer Ti_3_C_2_T_x_ nanosheets. Further analysis using TEM and SAED (Figure 16e,f) demonstrates the hexagonal crystal structure of the DMSO–Ti_3_C_2_T_x_ flakes. AFM measurements (Figure 16g) indicate that the thickness of single-layer DMSO–Ti_3_C_2_T_x_ is approximately 1.6 nm, consistent with the single-layer thickness reported by Lipatov et al. [62]. Notably, in contrast to Ti_3_C_2_T_x_ synthesized by traditional HF etching, DMSO–Ti_3_C_2_T_x_ does not disperse in pure water [63]. However, it achieves excellent dispersion in a DMSO–water mixed solution through simple stirring. As shown in Figure 16h, the etching rate of this method significantly outperforms other conventional aqueous or anhydrous synthesis methods, highlighting its exceptional efficiency [64,65,66,67,68,69]. Additionally, DMSO–Ti_3_C_2_T_x_ exhibits outstanding mechanical properties, with films fabricated from it achieving an ultimate tensile strength of 167 ± 8 MPa, attributed to the larger flake size.

**Figure 17:**
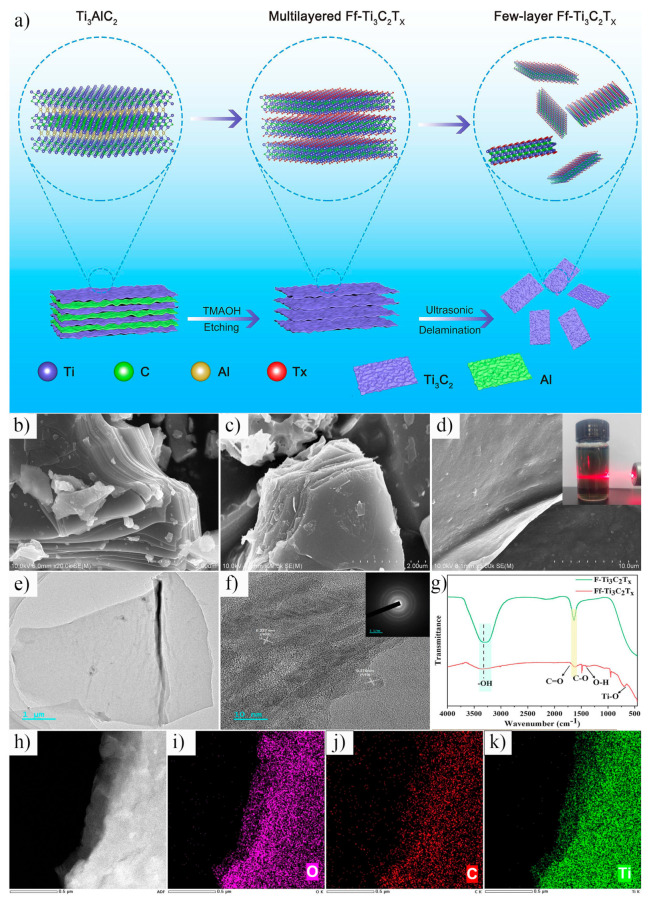
(**a**) Schematic diagram of etching process of Ff–Ti_3_C_2_T_x_; SEM images of (**b**) Ti_3_AlC_2_, (**c**) F–Ti_3_C_2_T_x_, and (**d**) Ff–Ti_3_C_2_T_x_; (**e**,**f**) TEM images of Ff–Ti_3_C_2_T_x_; (**g**) FTIR spectra of F–Ti_3_C_2_T_x_ and Ff–Ti_3_C_2_T_x_; (**h**–**k**) elemental mapping in Ff–Ti_3_C_2_T_x_ [70].

Wang et al. [70] demonstrated that tetramethylammonium hydroxide (TMAOH) solution can be used as an effective etchant for Ti_3_AlC_2_, successfully synthesizing fluorine-free Ti_3_C_2_T_x_. The Ti_3_C_2_T_x_ produced by this method exhibits abundant active sites and excellent hydrophilicity. The etching process is depicted in Figure 17a, and the corresponding chemical reaction is as follows:

Ti_3_AlC_2_ + OH^−^ + H_2_O → Ti_3_C_2_T_x_ + Al(OH)_3_ + H_2_ (17)

Figure 17b–d show the microscopic morphology of Ti_3_AlC_2_, HF-etched Ti_3_C_2_T_x_ (F-Ti_3_C_2_T_x_), and TMAOH-etched Ti_3_C_2_T_x_ (Ff–Ti_3_C_2_T_x_). The Ff–Ti_3_C_2_T_x_ exhibits a typical few-layer nanosheet morphology with a lateral size of approximately 15 μm, a layer thickness of ~0.6 nm, and a specific surface area of 17.37 m^2^/g. TEM analysis (Figure 17e,f) reveals lattice spacings of 0.227 nm and 0.270 nm for Ff–Ti_3_C_2_T_x_, corresponding to the (103) and (110) planes of Ti_3_C_2_, respectively, indicating a well-ordered crystal structure. Elemental mapping via TEM (Figure 17h–k) demonstrates that the O, C, and Ti are uniformly distributed across the Ff–Ti_3_C_2_T_x_ nanosheets, further confirming the structural consistency. Fourier transform infrared spectroscopy (FTIR) (Figure 17g) was employed to analyze the surface functional groups. The results indicate that compared to F–Ti_3_C_2_T_x_, Ff–Ti_3_C_2_T_x_ contains a greater abundance of oxygen-containing functional groups, such as C=O, C-O, O-H, and Ti-O. The presence of these groups greatly improves the hydrophilicity of Ff–Ti_3_C_2_T_x_, underscoring its versatility for various applications.

**Figure 18:**
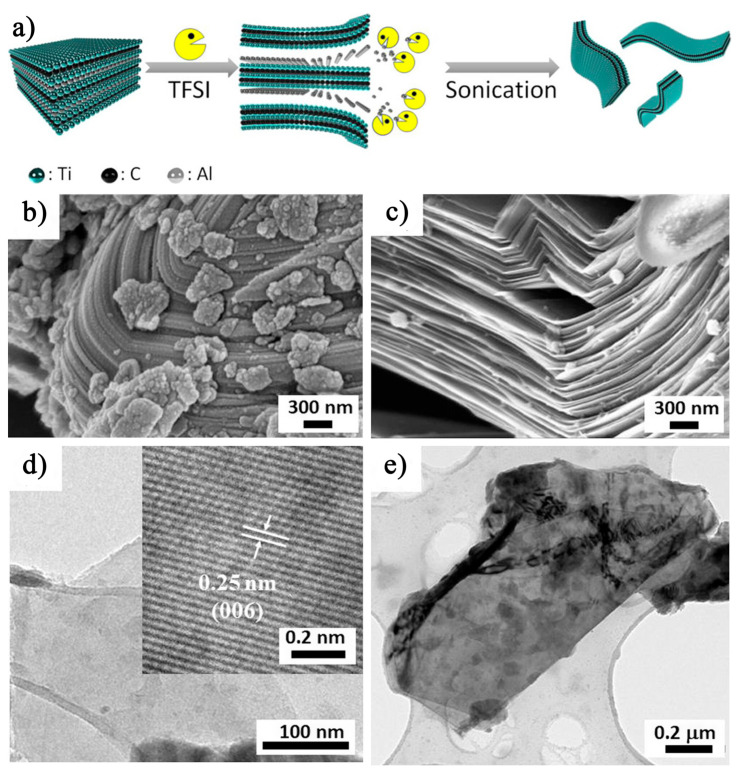
(**a**) Schematic illustration of MXene synthesis; (**b**) SEM images for Ti_3_AlC_2_ MAX powder; (**c**) monolayer; (**d**) TEM images for prepared Ti_3_C_2_T_x_ MXene; (**e**) thin layers [71].

In addition to the previously discussed etching methods, Chen et al. [71] introduced a novel approach in 2023 using Bis(trifluoromethanesulfonyl)imide (TFSI) as an etchant to selectively dissolve the Al layers in Ti_3_AlC_2_, thereby synthesizing Ti_3_C_2_T_x_ MXene nanosheets. The etching process is illustrated in Figure 18a. SEM images in Figure 18b,d show the morphology of Ti_3_AlC_2_ MAX before and after TFSI treatment, respectively. Prior to treatment, Ti_3_AlC_2_ MAX exhibits a distinct micron-scale layered structure, whereas the TFSI-treated Ti_3_C_2_T_x_ MXene displays a characteristic accordion-like morphology. TEM analysis (Figure 18c) further confirms that Ti_3_C_2_T_x_ MXene maintains a well-ordered crystal structure, with a lattice spacing of 0.25 nm corresponding to the (006) plane of Ti_3_C_2_. A key advantage of this method is that TFSI serves both as an etchant and as an intercalating agent, simplifying the experimental procedure while enhancing efficiency. This dual functionality streamlines the synthesis process, offering an effective and rational approach to producing high-quality Ti_3_C_2_T_x_ MXene.

The above content provides a comprehensive overview of various etching methods for Ti_3_C_2_T_x_ MXene, each offering unique advantages depending on specific requirements and experimental conditions. Table 1 summarizes the key etching parameters of different etching methods, facilitating the selection of the most suitable approach for different technical needs.

It is important to note that due to the abundance of active sites on the MXene surface, it is highly reactive with environmental substances like water and oxygen, resulting in alterations to the surface functional groups and, as a consequence, influencing the material’s properties. This issue is particularly prominent under humid or high-temperature conditions, where MXene is susceptible to oxidation or degradation, which can result in performance degradation. Therefore, Ti_3_C_2_T_x_ MXene typically cannot remain stable in solution for extended periods and is often required to be freshly prepared for immediate use, limiting its long-term storage and practical applications. To address this issue, researchers are exploring various methods to improve the stability of MXene, thereby enhancing its durability and extending its lifetime.

In 2022, Ashok et al. [87] improved the stability of Ti_3_C_2_T_x_ by treating it with NH_4_OH following etching Ti_3_AlC_2_ using an HCl and LiF solution. During this process, NH_4_OH reacted with the in situ generated HF, forming the buffer compound NH_4_F, which played a crucial role in regulating the oxidation behavior and exfoliation properties of Ti_3_C_2_T_x_. This modification enhanced the structural integrity and morphology of Ti_3_C_2_T_x_, leading to a superior antioxidant performance in air. Moreover, Ashok et al. observed that the Ti_3_C_2_T_x_ produced via in situ HF etching exhibited strong pH dependence in its colloidal solution. Under neutral pH conditions, the edges of Ti_3_C_2_T_x_ carried negative charges, while the surface exhibited positive charges, thus promoting stability. In acidic conditions, Ti_3_C_2_T_x_ underwent sedimentation, whereas in alkaline pH, flocculation occurred only when the pH exceeded 12. After NH_4_OH modification, the pH of the solution stabilized around 6, enabling Ti_3_C_2_T_x_ to remain stable for over 40 days without aggregation.

**Figure 19:**
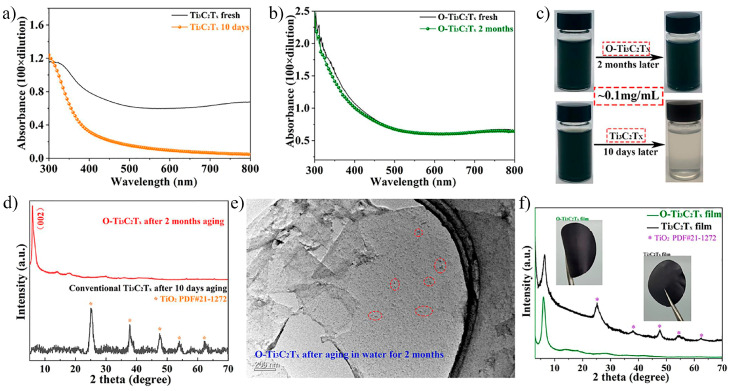
UV–Vis spectra of (**a**) traditional Ti_3_C_2_T_x_ and (**b**) O-Ti_3_C_2_T_x_; (**c**) aqueous suspensions and (**d**) XRD patterns of traditional Ti_3_C_2_T_x_ and O-Ti_3_C_2_T_x_; (**e**) TEM image of O-Ti_3_C_2_T_x_ after 2 months; (**f**) XRD patterns of Ti_3_C_2_T_x_ and O-Ti_3_C_2_T_x_ films after 2 months [88].

In 2023, Li et al. [88] treated Ti_3_C_2_T_x_, etched with LiF and HCl, using tetramethylammonium hydroxide (TMAOH). This treatment successfully removed etching by-products and increased the number of oxygen-containing functional groups on the Ti_3_C_2_T_x_ surface, significantly enhancing its durability and lifetime. After TMAOH treatment, the relative atomic ratio of fluorine in Ti_3_C_2_T_x_ decreased from 41.44% to 11.34%, while the oxygen content increased from 13.36% to 36.77%. When comparing the colloidal solution of Ti_3_C_2_T_x_ treated with TMAOH (O–Ti_3_C_2_T_x_) and traditional MXene colloid solutions (Figure 19a–d), it was found that traditional MXene solutions degraded through oxidation within 10 days, whereas O–Ti_3_C_2_T_x_ exhibited no significant degradation even after two months. Additionally, the microstructure of O–Ti_3_C_2_T_x_ remained largely unchanged after two months (Figure 19e), demonstrating superior stability compared to traditional MXene. Li et al. also found that O–Ti_3_C_2_T_x_ MXene films showed significantly better durability and lifetime in a controlled temperature and humidity environment compared to traditional Ti_3_C_2_T_x_ films. As shown in the XRD (Figure 19f), the traditional Ti_3_C_2_T_x_ film exhibited peaks corresponding to TiO_2_ after two months, indicating significant oxidation, while no such phenomenon was observed in the O–Ti_3_C_2_T_x_ film.

**Figure 20:**
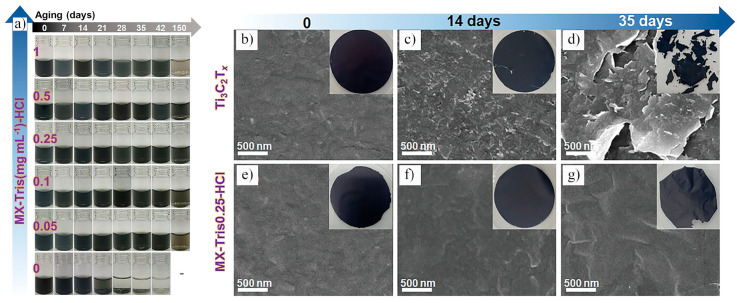
(**a**) Color change of Ti_3_C_2_T_x_ suspension; (**b**–**g**) aging process of Ti_3_C_2_T_x_ film [89].

In 2024, Tan et al. [89] employed Tris(hydroxymethyl)aminomethane (Tris)-HCl buffer solutions to stabilize Ti_3_C_2_T_x_ MXene dispersions. The Tris in the buffer formed Ti–N bonds with the Ti atoms on the surface of Ti_3_C_2_T_x_, effectively preventing oxidation and degradation. By adjusting the pH of the Tris-HCl buffer, they achieved enhanced durability and extended lifetime for Ti_3_C_2_T_x_ MXene dispersions. Experimental results (Figure 20a) showed that untreated Ti_3_C_2_T_x_ liquid exhibited noticeable color changes and oxidation after 14 days, whereas the Ti_3_C_2_T_x_ dispersion treated with Tris-HCl buffer exhibited significantly improved stability. Specifically, the Ti_3_C_2_T_x_ dispersion treated with 0.25 mg/mL Tris-HCl remained stable for 150 days. Moreover, oxidation degradation substantially affected the lateral dimensions of Ti_3_C_2_T_x_. Figure 20b–g show the surface state of Ti_3_C_2_T_x_ treated with 0.25 mg/mL Tris-HCl buffer and untreated Ti_3_C_2_T_x_. The untreated Ti_3_C_2_T_x_ displayed visible white boundaries after 14 days, indicating the onset of oxidation, which worsened by day 35, resulting in the fragmentation of the Ti_3_C_2_T_x_ sheets. In contrast, Ti_3_C_2_T_x_ treated with 0.25 mg/mL Tris-HCl maintained its surface morphology without significant changes. The Tris-HCl buffer not only prevented oxidation but also acted as an interlayer spacer, facilitating ion diffusion and improving the material’s electrochemical performance. After treatment, Ti_3_C_2_T_x_ films exhibited a capacitance of 212.4 F/g at a current density of 500 A/g, which remained at 156 F/g after 35 days, significantly higher than the 33.4 F/g observed for untreated Ti_3_C_2_T_x_ films.

## 3. Delamination

The accordion-like structure of MXene obtained through etching is not the final product and requires further delamination. Although the etching process effectively disrupts the interlayer chemical bonds in the MAX phases, relaxing the layered structure of MXene, van der Waals forces and strong bonds formed during etching may still exist in certain cases. These mechanical resistances hinder further delamination. As a result, additional measures are necessary to overcome these forces, expand the interlayer spacing, and facilitate successful delamination. Typically, delamination is achieved by introducing intercalants that penetrate and expand the interlayer structure of MXene, reducing interlayer interactions and enabling the separation of single-layer MXene sheets. Following the introduction of intercalants, ultrasonic or vibration treatment is commonly employed to accelerate the delamination process. Conventional delamination methods often use high-speed centrifugation, where centrifugal forces are applied to separate MXene layers. Centrifugation speeds are typically set above 3500 rpm to generate sufficient force for layer separation. However, excessively high speeds can lead to the breakage of MXene nanosheets, reducing their lateral size and compromising their structural integrity [25]. Therefore, the development of novel delamination techniques to improve efficiency has become a key area of research.

For Ti_3_C_2_T_x_ MXene, chloride salts are among the most commonly used intercalants. In 2016, Ghidiu et al. [41] employed a mixed solution of HF and LiCl to etch the Ti_3_AlC_2_ MAX phase. After etching, the sample was washed with 6 M HCl to remove the LiF precipitate. Subsequently, chloride salt solutions (LiCl, NaCl, KCl, RbCl, MgCl_2_ and CaCl_2_) were introduced for intercalation, resulting in a Ti_3_C_2_T_x_ MXene with an interlayer spacing of approximately 25 Å. Studies have shown that different cations exhibit varying intercalation effects on MXene. Moreover, due to its clay-like properties, MXene responds differently to humidity changes depending on the type of intercalant used.

**Figure 21:**
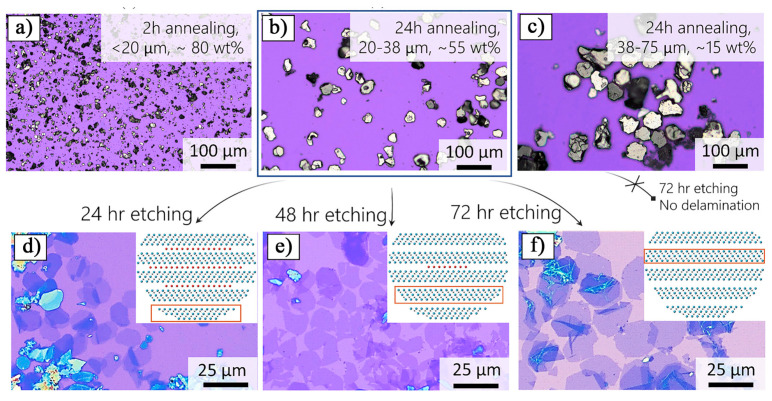
Optical micrographs of (**a**–**c**) MAX phase and (**d**–**f**) MXene at different etching time periods [90].

In 2022, Shekhirev et al. [90] proposed a soft delamination method using Li^+^ as an intercalant. In this approach, etched Ti_3_C_2_T_x_ MXene is immersed in a LiCl solution and stirred at 350 rpm for 18 h at 35 °C, yielding single-layer MXene nanosheets with a large surface area. This method not only prevents nanosheet fragmentation but also preserves the size of the original MAX phase particles. By employing large MAX particles as precursors, the resulting MXene exhibited lateral dimensions of up to 40 μm and layer thicknesses ranging from 2.5 to 3 nm. Figure 21 illustrates the microstructure of both the etched MAX phase and the delaminated MXene, demonstrating that the soft delamination method effectively preserved the size of the MAX phase flakes while minimizing structural damage. This method provides a promising foundation for the industrial application of MXene.

**Figure 22:**
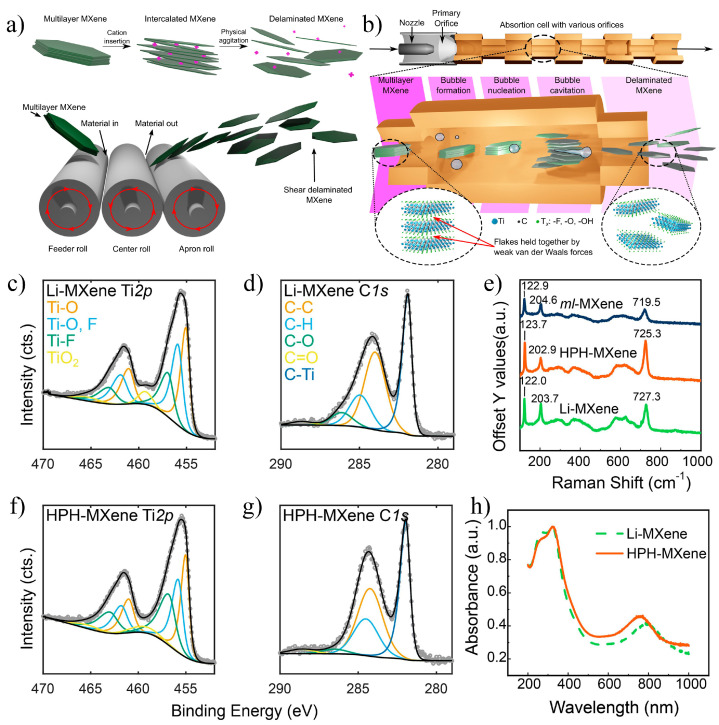
(**a**) Traditional layering method and three-roll mill layering method; (**b**) schematic diagram of HPH treatment; XPS of (**c**,**d**) Li-MXene and (**f**,**g**) HPH-MXene; (**e**) Raman spectra of MXene; (**h**) UV–Vis spectra of MXene [91,92].

While traditional chemical intercalation methods can achieve the delamination of multilayer MXene, these processes are time-consuming and generate substantial chemical waste. Additionally, intercalants can influence the properties of MXene, and some may be toxic, limiting their applications in various fields. To address these challenges, Inman et al. [91] proposed a physical delamination method in 2022, using a three-roll mill to shear multilayer MXene. This approach enables the delamination of single-layer or few-layer Ti_3_C_2_T_x_ MXene without the need for chemical intercalants. The process is illustrated in Figure 22a. By applying a shear rate exceeding 15,000 s^−1^, the van der Waals forces between layers are effectively weakened, allowing for successful delamination. This method streamlines the delamination process while eliminating residual chemical intercalants. The resulting Ti_3_C_2_T_x_ exhibited a capacitance of 337 F·g^−1^ in a 3 M H_2_SO_4_ electrolyte, comparable to that produced via LiCl intercalation. However, a small fraction of undelaminated layers remained in the product. Notably, this method is not limited to Ti_3_C_2_T_x_ and can be extended to V_2_CT_x_ MXene. The 3RM-V_2_CT_x_ prepared using this method demonstrated electrochemical performance in a 5 M LiCl electrolyte comparable to that of Li-V_2_CT_x_ prepared by ion exchange, highlighting the scalability and potential of the three-roll milling method for industrial MXene production.

In 2023, Inman et al. [92] introduced a high-pressure homogenization (HPH) method for delamination, which uses a combination of high shear, cavitation, and impact forces to effectively separate MXene layers without requiring post-treatment or chemical intercalants. This approach significantly enhances delamination efficiency compared to conventional methods. The schematic of the delamination process is shown in Figure 22b. As the MXene solution passes through the HPH unit, the reduced pore sizes subject the solution to intense shear forces. As the solution moves from a smaller nozzle to a larger one, sudden pressure changes induce bubble formation, which aids in the delamination of the accordion-like MXene structure. The resulting solution forms a colloidal suspension similar to that obtained through LiCl intercalation. Since MXene produced by HPH (HPH-MXene) lacks Li^+^ to neutralize surface charges, its Zeta potential is expected to be more negative than that of LiCl-etched MXene (Li-MXene). However, the measured Zeta potential of HPH-MXene is −27.4 ± 0.3 mV, slightly higher than the −31.3 ± 0.8 mV of Li-MXene. This discrepancy may be related to the particle size and solution stability, as MXene solutions with more single-layer nanoflakes typically exhibit higher Zeta potentials. Furthermore, Raman spectroscopy analysis (Figure 22c–h) revealed that HPH-MXene has lower oxidation levels and fewer defects than Li-MXene, further demonstrating that the HPH method produces higher-quality MXene compared to traditional chemical intercalation methods.

As MXene materials continue to demonstrate significant potential across various applications, evaluating the environmental impact of their synthesis processes has become increasingly important. To support the sustainable development of MXene, conducting a Life Cycle Assessment (LCA) is crucial. The synthesis of Ti_3_C_2_T_x_ MXene involves various etching methods, each with distinct environmental impacts, making LCA a crucial tool for systematically comparing these differences. Through LCA, a comprehensive comparison of the environmental impacts of commonly used synthesis methods can be made, providing valuable data to guide the optimization and environmentally friendly development of MXene synthesis processes.

**Figure 23:**
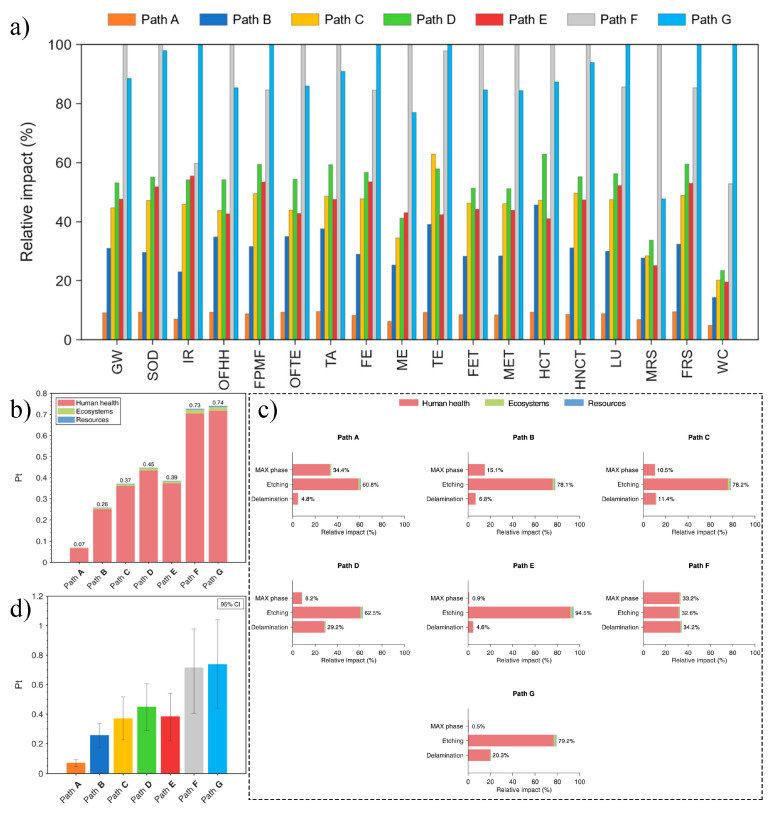
(**a**) Impact of different synthetic pathways on the environment; (**b**) single score results of different synthetic pathways; (**c**) influence of different steps in the synthesis of Ti_3_C_2_T_x_; (**d**) single score results for the synthesis Ti_3_C_2_T_x_ according to different synthesis pathways [93].

In 2024, Ungureanu et al. [93] conducted the first systematic comparison of the life cycle environmental impacts associated with seven different methods for synthesizing Ti_3_C_2_T_x_ MXene. The results, presented in Table 2 and Figure 23a, highlight the variations in environmental impact across these methods. To enable a more accurate comparison, the researchers applied normalization and weighted scoring methods. Based on the scoring results (Figure 23b), the environmental impact follows this order: pathway A < B < C < E < D < F < G, with human health being the most affected category, followed by ecosystem damage and resource consumption. Figure 23c illustrates the environmental impacts at various steps of Ti_3_C_2_T_x_ MXene synthesis, including MAX phase preparation, etching, and delamination. It is clear that the etching step has the most significant environmental impact, primarily due to its extended duration and high energy consumption. To further validate the reliability of their findings, Ungureanu et al. performed uncertainty analysis using Monte Carlo (MC) simulations, with the results shown in Figure 23d. The simulations revealed the variability within the 95% confidence interval. The analysis confirmed that pathway A, compared to the other six methods, exhibits the least environmental impact, consistent with the trend observed in Figure 23b, thus supporting the robustness of their conclusions. This comprehensive LCA not only identifies the environmental impacts of various synthesis methods but also provides strong data support for the environmental optimization of future MXene synthesis processes.

## 4. Applications of Ti_3_C_2_T_x_ MXene in Ceramics

Ceramic materials are highly valued for their exceptional strength, hardness, high-temperature resistance, and corrosion resistance, making them essential in applications such as aerospace, military protection, electronics, and energy systems [101,102,103]. However, the highly ordered atomic structure that contributes to these beneficial properties also renders ceramics prone to poor crack resistance and brittle fracture when subjected to external forces. This brittleness remains a significant challenge for the broader application of ceramics. To mitigate this limitation, researchers have explored various strategies, with the incorporation of second-phase materials emerging as one of the most effective solutions. In recent years, nanoscale second-phase materials such as carbon nanotubes (CNTs), graphene, nano-silicon nitride (Si_3_N_4_), nano-silicon carbide (SiC), and MXene have attracted considerable attention due to their excellent mechanical properties and unique nanoscale effects [104,105,106]. Among these, Ti_3_C_2_T_x_ MXene stands out due to its distinctive physicochemical properties, particularly its 2D nanostructure, high electrical conductivity, and exceptional mechanical performance. These advantages make Ti_3_C_2_T_x_ MXene a promising candidate for reinforcing ceramic matrix composites in applications like aerospace, military defense, and energy storage.

**Figure 24:**
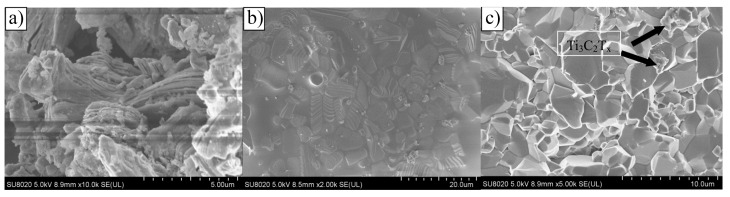
(**a**) Original morphology of Ti_3_C_2_T_x_; (**b**) morphology of sintered Ti_3_C_2_T_x_; (**c**) fracture surface morphology of Ti_3_C_2_T_x_–Al_2_O_3_ composites [107].

In 2017, Fei et al. [107] conducted pioneering research on incorporating Ti_3_C_2_T_x_ MXene as a reinforcing phase in bauxite, leading to the development of Ti_3_C_2_T_x_–Al_2_O_3_ composite ceramics through high-temperature sintering. Their study demonstrated that increasing the weight fraction of Ti_3_C_2_T_x_ in the Al_2_O_3_ matrix significantly enhanced several mechanical properties, including volume density, hardness, bending strength, and fracture toughness. Specifically, at a sintering temperature of 1500 °C, the Al_2_O_3_ composite ceramics containing 2 wt.% Ti_3_C_2_T_x_ exhibited approximately 300%, 150%, and 300% improvements in hardness, bending strength, and fracture toughness, respectively, compared to pure Al_2_O_3_ ceramics. Microstructural analysis, as shown in Figure 24, revealed that during high-temperature sintering, part of the Ti_3_C_2_T_x_ underwent melting (Figure 24b), while the majority retained its characteristic accordion-like structure [108]. Figure 24c illustrates how the melted MXene filled the pores within the Al_2_O_3_ matrix and was firmly anchored at the Al_2_O_3_ grain boundaries through interfacial interactions, significantly improving the relative density of the composite ceramics. Furthermore, the incorporation of Ti_3_C_2_T_x_ reduced the sintering temperature of Al_2_O_3_ ceramics and inhibited excessive grain growth, which is crucial for enhancing the mechanical properties of ceramic materials. Notably, Ti_3_C_2_T_x_ effectively hindered crack propagation, inducing cracks to follow more tortuous paths via the crack deflection mechanism, thereby relieving stress and markedly improving the fracture toughness of the composite ceramics.

**Figure 25:**
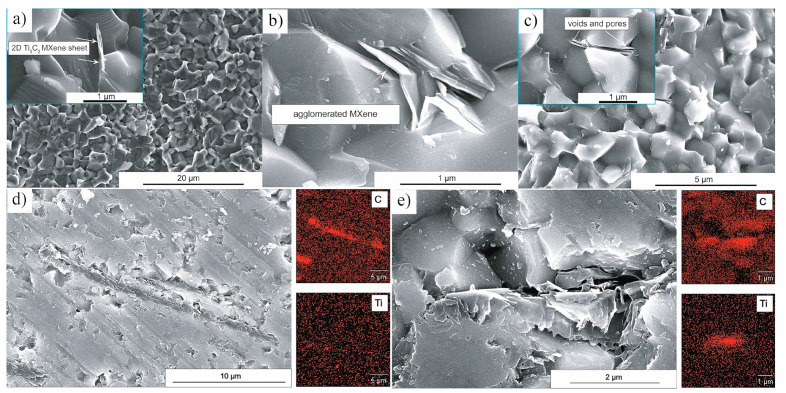
Fracture surface of SiC composites with (**a**) 0.7 wt.% Ti_3_C_2_T_x_, (**b**) 2.5 wt.% Ti_3_C_2_T_x_, and (**c**) 3.0 wt.% Ti_3_C_2_T_x_; (**d**,**e**) EDS of SiC composites with 2.0 wt.% Ti_3_C_2_T_x_ [109].

In 2021, Petrus et al. [109] explored the incorporation of Ti_3_C_2_T_x_ MXene into SiC powders and successfully fabricated Ti_3_C_2_T_x_–SiC composites through powder metallurgy at 1900 °C for 30 min. The effects of Ti_3_C_2_T_x_ incorporation on the microstructure and mechanical properties of the composites were systematically investigated. SEM images (Figure 25a–c) reveal that, at low Ti_3_C_2_T_x_ content, the interface between the Ti_3_C_2_T_x_ layers and the SiC matrix exhibited strong bonding with a well-formed, tight connection, showing no visible gaps or discontinuities. However, as the Ti_3_C_2_T_x_ content increased, noticeable voids appeared between the Ti_3_C_2_T_x_ layers and the SiC matrix, indicating a reduction in interface bonding strength. Further elemental analysis via EDS identified two distinct layered structures within the composite: one composed of thicker graphite layers (Figure 25d), and the other consisting of thinner C-Ti layers (Figure 25e). These layered structures effectively suppressed the growth of SiC grains, contributing to an improvement in the composite’s hardness. Compared to pure SiC ceramics (20.7  ±  0.4 GPa), the hardness of the SiC composite ceramic containing 1.5 wt.% Ti_3_C_2_T_x_ increased by over 10%, reaching 23.2  ±  0.4 GPa. Additionally, these layered structures hinder crack propagation through mechanisms such as crack deflection and bridging, significantly enhancing the fracture toughness of the composite material. Specifically, the fracture toughness of the SiC composite with 0.2 wt.% Ti_3_C_2_T_x_ increased by nearly 50%, from 3.1  ±  0.2 MPa·m^1/2^ in pure SiC ceramics to 4.5  ±  0.2 MPa·m^1/2^ in the composite.

**Figure 26:**
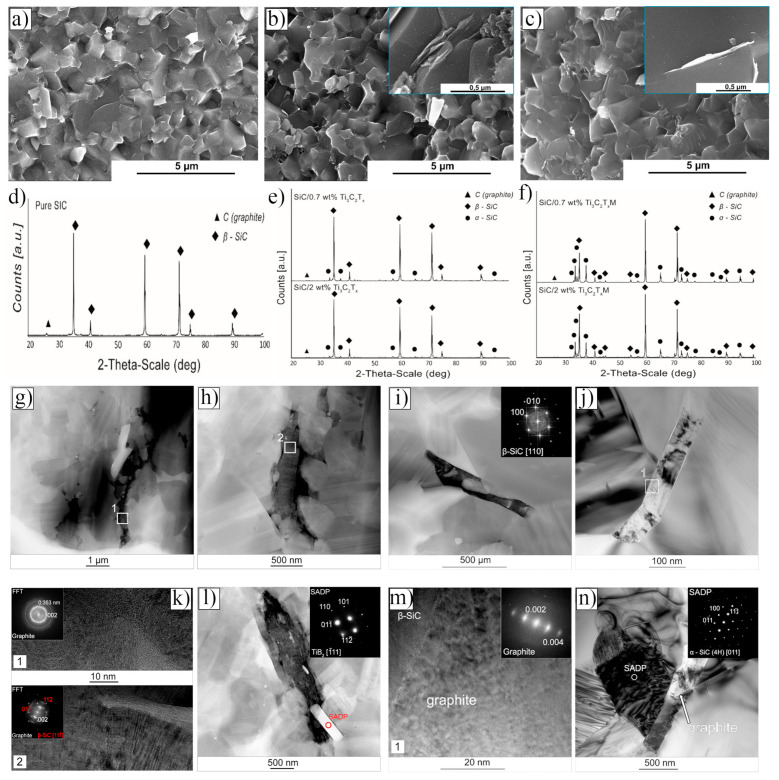
Fracture surface of (**a**) pure SiC ceramic, (**b**) SiC-1.5 wt.% Ti_3_C_2_T_x_ composites, and (**c**) SiC-1.5 wt.% Ti_3_C_2_T_x_M composites; phase composition of (**d**) pure SiC ceramic, (**e**) SiC-0.7 wt.% Ti_3_C_2_T_x_ composites, and (**f**) SiC-0.7 wt.% Ti_3_C_2_T_x_M composites; (**g**,**h**,**j**) TEM images of SiC-2 wt.% Ti_3_C_2_T_x_ composites; (**i**) HRTEM images of positions 1 and 2 in (**g**,**h**); (**k**,**l**,**n**) TEM images of SiC-2 wt.% Ti_3_C_2_T_x_ composites; (**m**) HRTEM images of position 1 in (**l**) [110].

In the same year, Petrus et al. [110] employed powder metallurgy to fabricate SiC composite ceramics reinforced with Ti_3_C_2_T_x_ and surface-modified Ti_3_C_2_T_x_ (Ti_3_C_2_T_x_M). The surface modification was achieved using a sol–gel method with a Y_2_O_3_/Al_2_O_3_ mixture. This study aimed to further explore the impact of surface modification on the microstructure and mechanical properties of composite ceramics. The fracture surface morphologies, shown in Figure 26a–c, reveal the presence of flaky structures at the grain boundaries in the MXene-reinforced SiC composites. Notably, compared to Ti_3_C_2_T_x_, the layered structure formed by Ti_3_C_2_T_x_M is considerably thinner, indicating that surface modification improves the dispersion and interface bonding of MXene within the composite. XRD analysis in Figure 26d shows that the addition of Ti_3_C_2_T_x_ alters the phase composition of the SiC composite ceramics, with Ti_3_C_2_T_x_M proving more effective in promoting the transformation from β-SiC to α-SiC. Further TEM analysis (Figure 26g–n) demonstrates that SiC composite ceramics containing Ti_3_C_2_T_x_M exhibit a more compact microstructure and fewer defects compared to those reinforced with Ti_3_C_2_T_x_. This improvement is attributed to the presence of Al_2_O_3_ and Y_2_O_3_ oxide layers on the surface of Ti_3_C_2_T_x_M, which react with the residual TiO_2_ on Ti_3_C_2_T_x_ surfaces to form a liquid phase during sintering. This liquid phase promotes the sintering process around the reinforcing phase, enhancing the interface quality and reducing the formation of internal defects, thus significantly improving the mechanical properties of the composite ceramics. The addition of MXene reinforcement significantly enhanced the fracture toughness of the composite ceramics compared to pure SiC. Specifically, the SiC composite containing 1 wt.% Ti_3_C_2_T_x_ exhibited the highest fracture toughness, reaching 5 MPa·m^1/2^, more than 50% higher than that of pure SiC ceramics and 15% higher than the SiC composite with 2.5 wt.% Ti_3_C_2_T_x_. These results suggest that surface-modified Ti_3_C_2_T_x_ reinforcement effectively enhances the mechanical properties of SiC composite ceramics, particularly in terms of fracture toughness.

**Figure 27:**
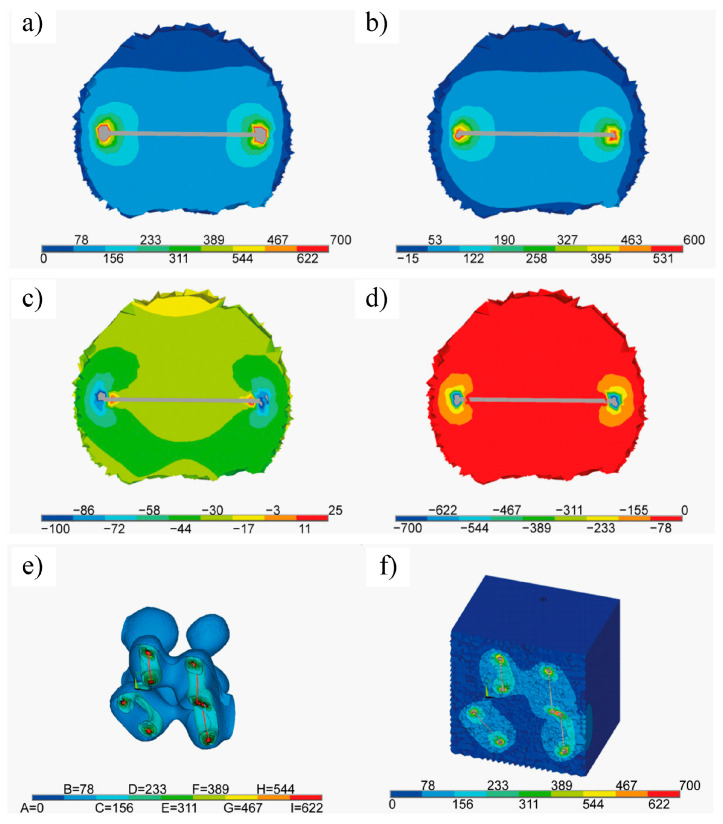
Stress state near flakes: (**a**) equivalent stress; (**b**) S1; (**c**) S2; and (**d**) S3; Von Mises equivalent stresses at the cross-section: (**e**) isosurfaces and (**f**) distribution [111].

As a reinforcing phase, Ti_3_C_2_T_x_ can significantly influence the residual stress levels and stress distribution within the matrix material. In 2022, the Petrus team [111] employed finite element analysis to explore the impact of Ti_3_C_2_T_x_ on the stress state in SiC composite ceramics. Figure 27 shows the distribution of equivalent stress and principal stresses (S1, S2, and S3) in regions near the Ti_3_C_2_T_x_ layers within the SiC composite. The results show that the edge regions of the Ti_3_C_2_T_x_ layers experience high compressive stress, with values approaching 700 MPa. The compressive stress fields play a crucial role in enhancing the material’s fracture toughness, as compressive stress helps close cracks and induces crack deflection, thereby preventing crack propagation.

As research into MXene as a reinforcing phase in ceramic materials continues to advance, a deeper understanding of this 2D nanomaterial has emerged. Studies have shown that MXene is highly susceptible to decomposition in air at temperatures as low as 200 °C, though its stability improves in inert environments such as argon. However, when exposed to temperatures exceeding 800 °C, MXene can undergo phase transformations, forming anatase. At temperatures above 1000 °C, MXene will completely oxidize, transitioning into rutile [112,113]. This thermal instability presents a challenge in the preparation of ceramic materials, which often requires high-temperature sintering processes. During sintering, MXene sheets are particularly vulnerable to oxidation and degradation, a phenomenon that is even more pronounced in single-layer MXene. Consequently, maintaining the thermal stability of MXene in high-temperature environments has become an urgent issue to address [114].

**Figure 28:**
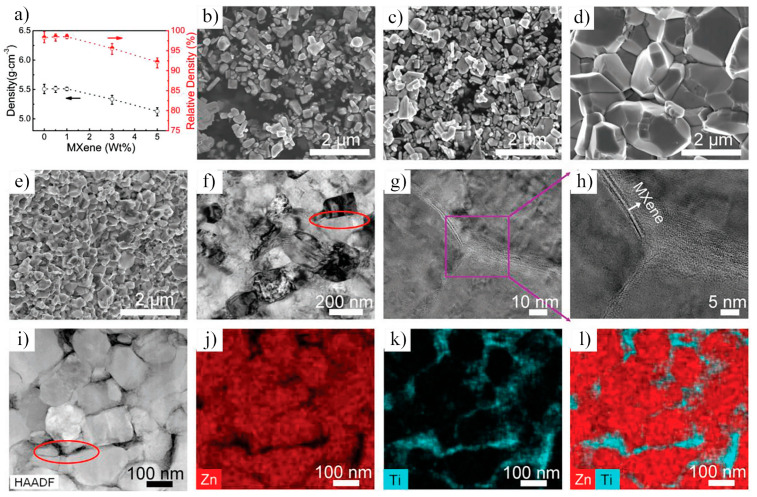
(**a**) Density and relative density of ZnO–Ti_3_C_2_T_x_ composites; (**b**) morphology of (**b**) ZnO and (**c**) ZnO–Ti_3_C_2_T_x_ raw powders; morphology of (**d**) ZnO ceramics and (**e**) ZnO–Ti_3_C_2_T_x_ composites; (**f**–**i**) TEM and (**j**–**l**) EDS of ZnO–Ti_3_C_2_T_x_ composites [115].

To address this challenge, in 2018, Guo et al. [115] employed the cold sintering process (CSP) to successfully co-sinter Ti_3_C_2_T_x_ MXene with ZnO ceramics, producing ZnO–Ti_3_C_2_T_x_ nanocomposites at low temperatures and effectively preventing the oxidation of Ti_3_C_2_T_x_. As shown in Figure 28a, the relative density of the ZnO–Ti_3_C_2_T_x_ composites prepared by this method exceeded 90%. Furthermore, SEM images comparing the original powders and the sintered bodies (Figure 28b–e) clearly reveal that the addition of Ti_3_C_2_T_x_ significantly suppressed grain growth in ZnO ceramics. TEM characterization results (Figure 28f–l) further demonstrate that the CSP technique effectively prevents diffusion between Ti_3_C_2_T_x_ and the ZnO ceramic matrix, allowing Ti_3_C_2_T_x_ to be uniformly distributed along the ZnO grain boundaries, thus preventing grain coarsening. Additionally, the study revealed that as the Ti_3_C_2_T_x_ content increased, the electrical conductivity of the ZnO–Ti_3_C_2_T_x_ nanocomposites increased by 1–2 orders of magnitude. Nanocomposites containing 0.5 wt.% Ti_3_C_2_T_x_ exhibited a 40–50% improvement in hardness and elastic modulus compared to pure ZnO ceramics. When the Ti_3_C_2_T_x_ content reached 5 wt.%, the hardness and elastic modulus increased by over 150%. These results demonstrate that the CSP technique offers an effective route for the preparation of 2D MXene–ceramic composites.

**Figure 29:**
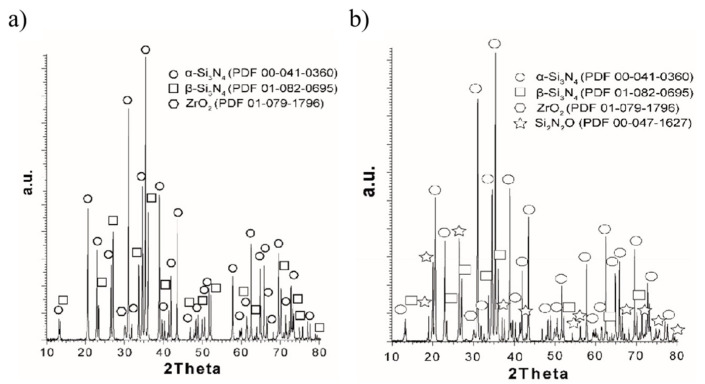
The XRD patterns of: (**a**) Si_3_N_4_ ceramics; (**b**) Si_3_N_4_–Ti_3_C_2_T_x_ composites [10].

In 2020, Wozniak et al. [10] successfully employed SPS technology to prepare Si_3_N_4_ ceramics reinforced with Ti_3_C_2_T_x_ MXene. SPS technology offers numerous advantages, including rapid heating rates, short sintering times, low sintering temperatures, quick cooling, and minimal holding times, all of which help reduce MXene decomposition during the sintering process. The lower sintering temperatures and rapid sintering process also play a crucial role in controlling the grain growth of Si_3_N_4_ ceramics, thereby further enhancing their mechanical properties. The study found that the incorporation of MXene significantly improved the hardness and fracture toughness of the Si_3_N_4_ ceramics. Moreover, XRD analysis presented in Figure 29 showed that the addition of MXene modified the phase transformation of Si_3_N_4_, facilitating the transition from α-Si_3_N_4_ to β-Si_3_N_4_ and introducing a new Si_2_N_2_O phase. These phase changes not only altered the ceramic’s phase composition but also had a significant impact on its mechanical properties.

**Figure 30:**
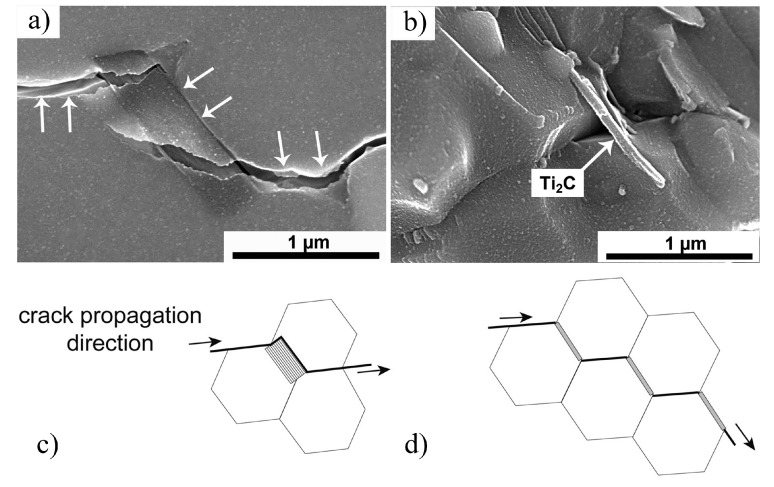
Crack propagation behavior for (**a**) non-delaminated Ti_2_C MXene and (**b**) delaminated 2D Ti_2_C MXene; crack propagation scheme for (**c**) non-delaminated Ti_2_C MXene and (**d**) delaminated 2D Ti_2_C MXene [116].

Similarly, Wozniak et al. [116] utilized the SPS process to prepare SiC–x/Ti_2_C composite ceramics (where x = 0.2, 0.5, 0.7, 1, 1.5, 2, 2.5, and 3 wt%) reinforced with Ti_2_C MXene. The results showed that the inclusion of Ti_2_C significantly enhanced the relative density, hardness, and fracture toughness of the SiC ceramics compared to pure SiC. Among the composites, those containing 1.5 wt.% Ti_2_C exhibited the best overall performance. The study demonstrated that the presence of MXene facilitated material transport during the sintering process, improving the relative density of the composite ceramics. As the MXene content increased, the SiC grain size gradually decreased, further boosting the mechanical properties. Crack propagation behavior, shown in Figure 30, revealed that delaminated 2D MXene as a reinforcement phase was particularly effective at hindering crack propagation. This mechanism forced the composite ceramics to absorb more energy during fracture. Moreover, crack bridging was observed in SiC composites containing delaminated MXene, which significantly contributed to the improvement in fracture toughness.

**Figure 31:**
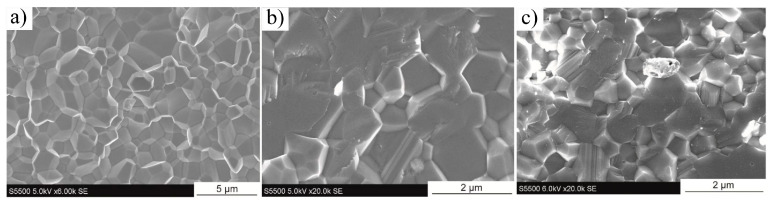
Fracture surface morphology of Al_2_O_3_ composite ceramics with: (**a**) Ti_3_C_2_; (**b**) Ti_3_C_2_–Ti; and (**c**) Ti_3_C_2_–Mo [117].

In 2021, Cygan et al. [117] proposed a structural modification strategy to prevent the oxidation and degradation of Ti_3_C_2_T_x_ MXene at high temperatures. They achieved this by sputtering thin Ti or Mo metal layers onto the surface of Ti_3_C_2_T_x_. In the Ti_3_C_2_T_x_–Al_2_O_3_ composite ceramics, the Ti/Mo metal layer served as a protective barrier, effectively preventing atomic migration and reorganization, thus limiting the diffusion of external environments and significantly altering the degradation process. The SEM images, shown in Figure 31, reveal that the Ti_3_C_2_Tx–Al_2_O_3_ composite ceramics exhibited a dense microstructure. Compared to Al_2_O_3_ composites with unmodified Ti_3_C_2_T_x_, the composites with modified Ti_3_C_2_T_x_ displayed a noticeable shift in fracture mode, transitioning from intergranular fracture to a mixed fracture mode, where both intergranular and transgranular fractures coexisted. Regarding mechanical properties, the composite ceramics with modified Ti_3_C_2_T_x_ demonstrated significant improvements. Specifically, the hardness and fracture toughness of the Al_2_O_3_ composites containing 0.5 wt.% modified Ti_3_C_2_T_x_ increased by 10% and 15%, respectively, compared to those containing unmodified Ti_3_C_2_T_x_.

In addition to enhancing the mechanical properties of ceramics, the incorporation of Ti_3_C_2_T_x_ MXene also significantly influences other critical aspects, including electromagnetic shielding, antioxidant properties, corrosion resistance, and fluorescence characteristics.

**Figure 32:**
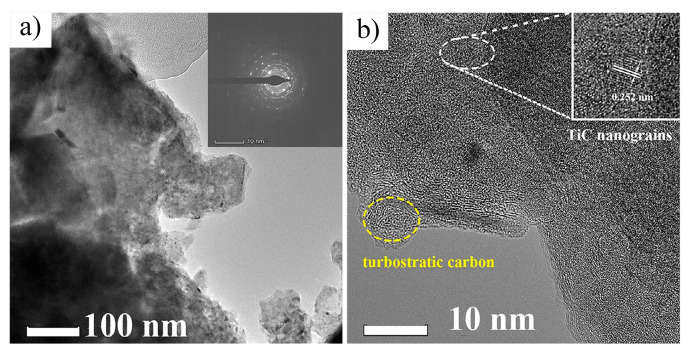
(**a**) TEM image of HRTEM of TiC/SiBCN ceramics; (**b**) TEM image of TiC nanoparticles [118].

In 2021, Ding et al. [118] introduced Ti_3_C_2_T_x_ MXene into polyborosilazane (SiBCN) to prepare TiC/SiBCN nanocomposite ceramics with exceptional electromagnetic absorption properties through pyrolysis treatment. TEM images shown in Figure 32 indicate that a substantial amount of TiC nanoparticles formed during the pyrolysis process. The formation of TiC nanoparticles not only enhanced the interface polarization but also provided a medium for the multiple reflections of electromagnetic waves. Additionally, the inherent defects in the TiC particles further boosted the dipole polarization effect. Together, these mechanisms collectively contributed to a significant enhancement in the electromagnetic absorption performance of composite ceramics. Remarkably, the TiC/SiBCN composites containing 5 wt.% Ti_3_C_2_T_x_ MXene achieved a minimum reflection coefficient of −45.44 dB at 10.93 GHz and demonstrated an effective absorption bandwidth spanning nearly the entire X-band, from 8.35 to 12.40 GHz. Furthermore, the study revealed that these ceramics exhibited excellent thermal stability, maintaining consistent electromagnetic absorption performance at temperatures up to 600 °C. This makes Ti_3_C_2_T_x_-infused SiBCN ceramics a promising material for advanced applications in radar and communication systems, where high-frequency absorption is crucial.

**Figure 33:**
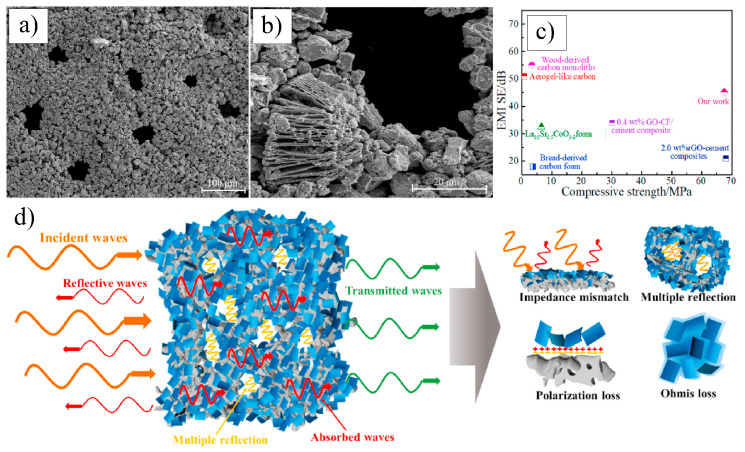
Surface morphology of Ti_3_C_2_T_x_/DFPS composites under (**a**) low magnification and (**b**) high magnification; (**c**) EMI shielding performance and compressive strength of different materials; (**d**) electromagnetic shielding mechanism of Ti_3_C_2_T_x_/DFPS composites [119].

In 2022, Lyu et al. [119] successfully developed Ti_3_C_2_T_x_/diatom frustule-derived porous silica (DFPS) composites (DM), which exhibited exceptional mechanical properties and outstanding electromagnetic interference (EMI) shielding performance. This was achieved by immersing DFPS ceramics in a Ti_3_C_2_T_x_ solution followed by annealing in an argon atmosphere. Figure 33a,b show the surface morphology of the Ti_3_C_2_T_x_ /DFPS composites, illustrating that Ti_3_C_2_T_x_ uniformly coated the DFPS framework, forming an efficient conductive network that enhances the material’s ability to absorb electromagnetic waves. The electromagnetic shielding mechanism of the Ti_3_C_2_T_x_/DFPS composite, depicted in Figure 33d, is primarily driven by absorption shielding. Specifically, the porous, layered Ti_3_C_2_T_x_ coating on the DFPS framework significantly improves the reabsorption and reflection efficiency of electromagnetic waves. Moreover, the impedance mismatch between Ti_3_C_2_T_x_ and the DFPS surface promotes the reflection and scattering of electromagnetic waves, simultaneously improving the material’s overall absorption capacity. Figure 33c compares the compressive strength and EMI shielding performance of various materials, demonstrating that the Ti_3_C_2_T_x_/DFPS composite achieves an optimal balance between mechanical strength and shielding effectiveness.

**Figure 34:**
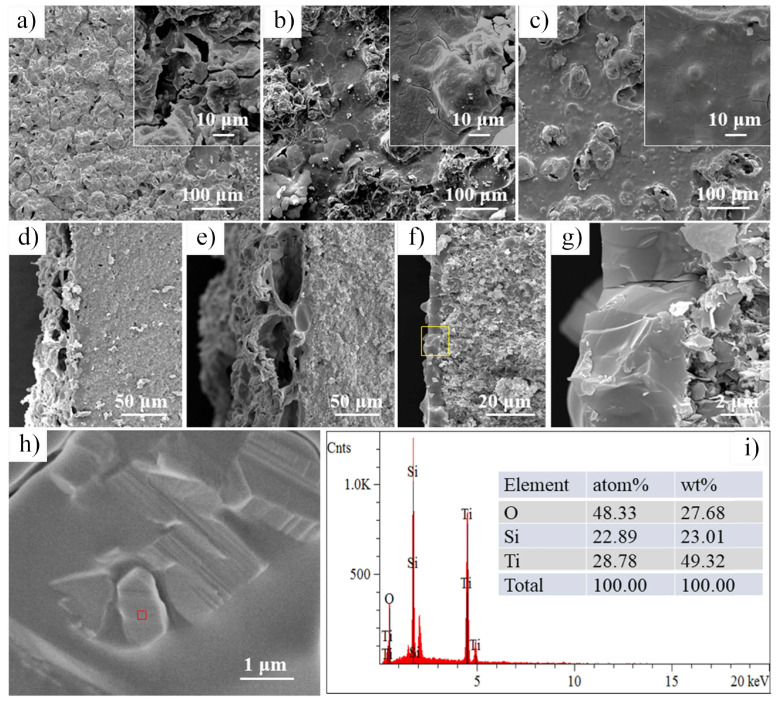
Surface and cross-section morphologies of Si–B–C–N ceramics with (**a**,**d**) 1.0 wt.%, (**b**,**e**) 1.5 wt.%, and (**c**,**f**) 3.0 wt.% Ti_3_C_2_T_x_ at 1100 °C; (**g**) is an enlarged image of the yellow rectangular box marked area in (**f**); (**h**) is a partial enlarge-ment of (**c**); (**i**) is the element analysis of the red rectangular area in (**h**) [120].

In 2022, Liang et al. [120] incorporated Ti_3_C_2_T_x_ MXene into quaternary Si–B–C–N ceramics to investigate its impact on the material’s oxidation resistance. As shown in Figure 34a–f, a dense oxide layer formed on the surface of the Si-B-C-N ceramics at 1100 °C. With increasing Ti_3_C_2_T_x_ content, from 1.0 wt.% to 3.0 wt.%, the thickness of the oxide layer decreased from 1.4 μm to 0.8 μm, indicating that Ti_3_C_2_T_x_ enhanced the oxidation resistance of the Si-B-C-N ceramics. However, when the temperature increased to 1600 °C, the oxidation of the Si-B-C-N ceramics significantly accelerated (Figure 34a–g). Specifically, Si–B–C–N ceramics with 1.0 wt.% and 1.5 wt.% Ti_3_C_2_T_x_ exhibited severe blistering on their surface, whereas those with 3.0 wt.% Ti_3_C_2_T_x_ maintained a dense and intact oxide layer. The results in Figure 34h,i show that at elevated temperature, TiO_2_ particles embedded within the SiO_2_ oxide layer created a pinning effect that effectively hindered the diffusion of SiO_2_, preventing further oxidation of the internal material. This antioxidant behavior is critical for extending the lifespan and performance of ceramic materials in high-temperature and oxidative environments, such as in aerospace and defense applications.

**Figure 35:**
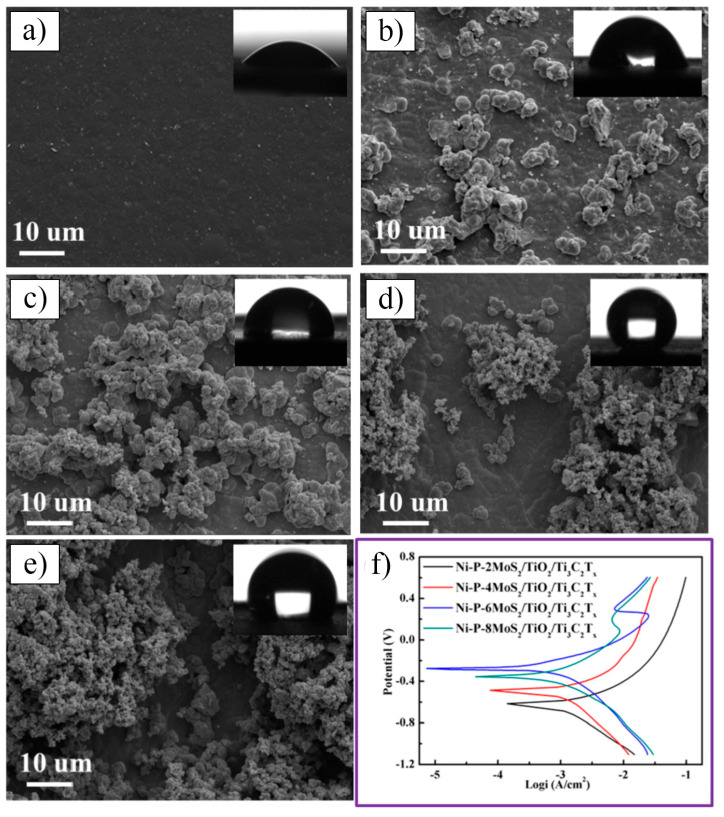
(**a**) Surface morphology of Ni–P coatings; surface morphology of (**b**) 2 g·L^−1^, (**c**) 4 g·L^−1^, (**d**) 6 g·L^−1^, and (**e**) 8 g·L^−1^ Ni–P–Ti_3_C_2_T_x_@TiO_2_/MoS_2_ coatings (inset with images of water drop); (**f**) Ra results of these composite coatings [121].

In 2020, Du et al. [121] synthesized Ti_3_C_2_T_x_ particles coated with TiO_2_ and MoS_2_ (Ti_3_C_2_T_x_@TiO_2_/MoS_2_) using a hydrothermal method. These particles were incorporated into Ni–P coatings via electroplating, significantly improving the corrosion resistance of the coatings. The incorporation of Ti_3_C_2_T_x_@TiO_2_/MoS_2_ particles modified the microstructure of the composite coating. Figure 35a–e show the surface morphology of Ni–P coatings with varying concentrations of Ti_3_C_2_T_x_@TiO_2_/MoS_2_ particles. It was observed that the surface of the Ni–P coating without Ti_3_C_2_T_x_@TiO_2_/MoS_2_ particles was relatively smooth. However, as the concentration of Ti_3_C_2_T_x_@TiO_2_/MoS_2_ particles increased, the surface became rougher, primarily due to the excellent conductivity of the Ti_3_C_2_T_x_@TiO_2_/MoS_2_ particles, which facilitated the formation of larger crystals within the coating. The inset in the top-right corner indicates that the addition of Ti_3_C_2_T_x_@TiO_2_/MoS_2_ particles significantly altered the wettability of the coating, making it more hydrophilic, with a maximum contact angle of 120°. This increase in contact angle contributed to enhanced corrosion resistance. Moreover, the Ti_3_C_2_T_x_@TiO_2_/MoS_2_ particles acted as cathodes within the coatings, enhancing the polarization of the anode. As shown in Figure 35f, when the concentration of Ti_3_C_2_T_x_@TiO_2_/MoS_2_ particles reached 6 g/L, the composite coating exhibited the lowest corrosion current and the highest polarization resistance, demonstrating optimal corrosion resistance.

**Figure 36:**
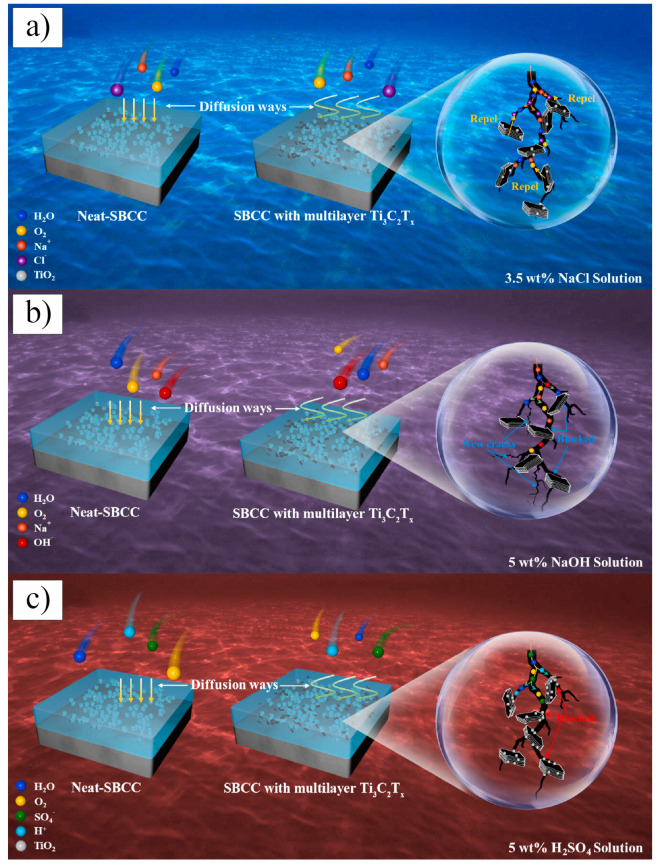
Anti-corrosion mechanism for the Ti_3_C_2_T_x_ reinforced SBCC in: (**a**) 3.5 wt.% NaCl; (**b**) 5 wt% NaOH; and (**c**) 5 wt% H_2_SO_4_ [122].

In 2023, Guan et al. [122] examined the impact of Ti_3_C_2_T_x_ MXene on the corrosion resistance of silicate-bonded ceramic coatings (SBCC). Pure SBCC exhibits poor physical barrier properties, allowing corrosive agents like water and oxygen to penetrate directly, limiting their corrosion resistance. In contrast, the addition of Ti_3_C_2_T_x_ significantly enhanced the structural integrity of SBCC, effectively preventing the diffusion of corrosive agents and substantially improving its corrosion resistance. The experimental results showed that SBCC with 1.6 wt.% Ti_3_C_2_T_x_ exhibited exceptional corrosion resistance in 3.5 wt.% NaCl, 5 wt.% NaOH, and 5 wt.% H_2_SO_4_ solutions, as shown in Figure 36. The most remarkable corrosion resistance was observed in 5 wt.% H_2_SO_4_ solution, where the impedance modulus of 3.45 × 10^6^ Ω·cm^2^ was approximately 79% higher than that of pure SBCC. Furthermore, the corrosion current density was 1.18 × 10^−8^ A/cm^2^, approximately one order of magnitude lower than that of pure SBCC.

**Figure 37:**
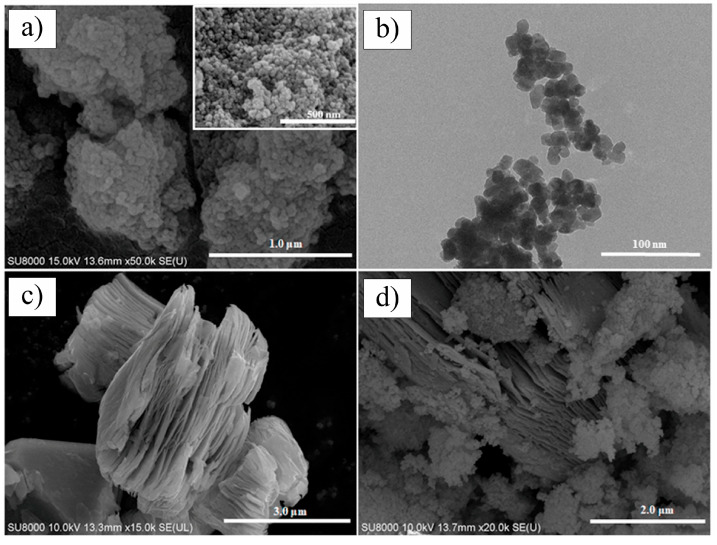
Microstructure of (**a**,**b**) 8YSZ: Eu^3+^; (**c**) MXene; and (**d**) MXene/8YSZ: Eu^3+^ [123].

In the same year, Dong et al. [123] explored the impact of Ti_3_C_2_T_x_ MXene on the fluorescence performance of Eu^3+^-doped 8 wt.% yttria-stabilized zirconia (8YSZ: Eu^3+^) powder. Microstructural analysis of the powder, as shown in Figure 37, reveals that the addition of Ti_3_C_2_T_x_ does not significantly alter the crystal structure of the 8YSZ: Eu^3+^ powder. However, some 8YSZ: Eu^3+^ particles are encapsulated by Ti_3_C_2_T_x_ flakes, while others are embedded between them. When compared to pure 8YSZ: Eu^3+^ powder, the luminescent intensity of the MXene/8YSZ: Eu^3+^ composites is markedly higher. This enhancement suggests that Ti_3_C_2_T_x_ MXene plays a crucial role in improving the optical properties of ceramic materials, which is particularly advantageous for applications requiring enhanced fluorescence or luminescence, such as in sensors and lighting technologies.

These studies further emphasize the multifunctional advantages of Ti_3_C_2_T_x_ MXene in enhancing the properties of ceramic materials. By incorporating Ti_3_C_2_T_x_, ceramics can exhibit not only improved mechanical properties but also enhanced electromagnetic absorption, oxidation resistance, corrosion resistance, and optical properties such as fluorescence. These improvements significantly expand the potential applications of ceramic materials in high-performance fields, including aerospace, military protection, electronics, and advanced coatings. The unique properties of Ti_3_C_2_T_x_ MXene make it a promising candidate for further research and development in advanced ceramic composites.

## 5. Summary and Outlook

This review summarizes the preparation methods of Ti_3_C_2_T_x_ MXene and its research progress in ceramic materials. As a novel 2D nanomaterial, Ti_3_C_2_T_x_ MXene has become a significant research focus in the field of material science, owing to its unique layered structure and excellent electrical, thermal, mechanical, and chemical properties. Ti_3_C_2_T_x_ MXene shows great promise, particularly in enhancing and functionalizing ceramic materials, offering remarkable potential.

In the preparation of Ti_3_C_2_T_x_ MXene, HF etching remains the most common and well-established method due to its simplicity and high yield. However, HF etching poses potential environmental and health hazards, and the -F functional groups on the surface of the Ti_3_C_2_T_x_ can negatively impact its subsequent applications. As a result, F-free or low-toxicity etching alternatives have become a research hotspot in recent years. Techniques such as acid–salt composite etching, alkaline etching, and molten salt etching have gained attention, as these methods can reduce environmental pollution while improving the quality and yield of Ti_3_C_2_T_x_. Despite progress in laboratory settings, challenges remain for large-scale production, particularly in terms of process stability, cost control, and scalability. In the future, composite etching methods that combine multiple chemical approaches may offer the most promising solution, effectively balancing environmental sustainability with the quality of Ti_3_C_2_T_x_.

The delamination process of Ti_3_C_2_T_x_ is crucial for producing high-quality single-layer or few-layer Ti_3_C_2_T_x_ nanosheets. Traditional chemical delamination methods typically rely on intercalants, but the use of such intercalants can not only negatively impact the performance of Ti_3_C_2_T_x_ but also generate substantial chemical waste. In recent years, advances in novel physical delamination technologies have made it possible to achieve effective delamination of Ti_3_C_2_T_x_ and produce high-quality nanosheets without the need for intercalants. By optimizing delamination equipment, controlling delamination conditions, and integrating advanced auxiliary delamination techniques, the delamination process for Ti_3_C_2_T_x_ can become more efficient and environmentally friendly, thus promoting its widespread application.

In the application of ceramic materials, Ti_3_C_2_T_x_ MXene, as a reinforcement phase, has demonstrated the ability to significantly enhance the mechanical properties, electromagnetic absorption, oxidation resistance, and corrosion resistance of ceramic materials. To address the instability of Ti_3_C_2_T_x_ at high temperatures, researchers have proposed using novel sintering techniques, such as CPS and SPS, to prepare Ti_3_C_2_T_x_–ceramic matrix composites with favorable outcomes. However, Ti_3_C_2_T_x_ MXene still faces several challenges in its application to ceramics. First, the large-scale synthesis of high-quality Ti_3_C_2_T_x_ remains a significant hurdle. Second, issues related to the dispersion of Ti_3_C_2_T_x_ and its compatibility with the ceramic matrix have not been fully resolved, which limits its widespread application. To address these challenges, future research should focus on the following areas: enhancing the surface functionalization of Ti_3_C_2_T_x_ to improve its compatibility with ceramic matrices, developing new delamination methods to prevent aggregation of Ti_3_C_2_T_x_ within the ceramic matrix, and further optimizing sintering processes, particularly to enhance the stability of Ti_3_C_2_T_x_ under high-temperature conditions.

Looking toward future research directions, the challenges in applying Ti_3_C_2_T_x_ MXene in ceramic materials will mainly be focused on large-scale synthesis, surface functionalization, and optimization of sintering processes. Future research could make breakthroughs in the following practical aspects: (1) developing more economical, environmentally friendly, and efficient synthesis processes, particularly focusing on the optimization and commercialization of F-free etching methods; (2) investigating surface modification and functionalization techniques to improve the dispersion and interfacial bonding of Ti_3_C_2_T_x_ with ceramic matrices, thereby enhancing its reinforcement effects; (3) exploring the impact of different sintering techniques on the properties of Ti_3_C_2_T_x_-ceramic composites, especially in terms of their stability and mechanical performance at high temperatures.

In conclusion, Ti_3_C_2_T_x_ MXene, as a highly promising 2D material, holds great potential for applications in ceramic materials. With continuous innovations in synthesis technologies, improvements in functionalization methods, and optimizations in sintering processes, Ti_3_C_2_T_x_ MXene is expected to become an ideal choice for enhancing the performance of ceramic materials. This will drive the development of ceramics toward higher performance and multifunctionality, promoting their applications in various practical fields.

## Figures and Tables

**Table 1:** The representative synthesis methods for Ti_3_C_2_T_x_ MXene.

Etching Methods	Etchant	Etching Environment	Ref.	Advantages	Disadvantages
Acid etching	50% HF	Room temperature, 24 h	[72]	①Simple process, easy to operate; ②Effectively removes Al or Si layers from MAX phase, resulting in high-purity Ti_3_C_2_T_x_ MXene.	①HF solution is highly corrosive, posing safety risks; ②Waste liquid treatment is challenging, with significant environmental impact; ③Surface rich in fluorine functional groups, which may reduce chemical activity or lead to instability in performance.
	49% HF	50 °C, 36 h	[73]		
	48% HF	Room temperature, 24 h	[74]		
	40% HF	40 °C, 30 h	[75]		
	50% HF	25 °C, 24 h	[76]		
	40% HF	25 °C, 24 h	[77]		
Acid–salt composite etching	50 mL 6 M HCl + NH_4_F	50 °C, 24 h	[78]	①HF is generated in situ, reducing the safety and environmental risks of directly using HF; ②Reduces the content of fluorine functional groups, enhancing chemical stability and electrochemical performance; ③The introduction of salt improves etching efficiency and uniformity of the product quality.	①Still an acidic system, environmental impact is not fully eliminated; ②High process complexity, requiring precise control of the acid and salt ratio and reaction conditions.
	4 g LiF + 50 mL 9 M HCl	Room temperature, 200 rpm, 72 h	[79]		
	12 M LiF + 9 M HCl	Room temperature, 24 h	[80]		
	6.0/7.5/9.0/12.0 M LiF + 9.0 M HCl	Room temperature, 300 rpm, 24 h	[81]		
	2 g LiF + 40 mL HCl (9.0 mol·L^−1^)	35 °C, 30 h	[82]		
	2 g NaF + 40 mL HCl (12 M)	60 °C, 48 h	[83]		
	0.8 g LiF + 10 mL 9 M HCl	Room temperature, 48 h	[84]		
Alkaline etching	30 mL 10 M NaOH	Room temperature, 1–5 day	[85]	①Generates fluorine-free terminated MXene, improving hydrophilicity and environmental friendliness; ②High etching efficiency, short reaction time.	①Requires high-concentration alkaline solution, high temperature, and long reaction times, leading to high energy consumption and increased safety risks; ②May generate alkaline waste liquid, requiring proper disposal to prevent environmental pollution
	50 mL 30%NaOH	100 °C, 24 h	[86]		
	50 mL 7 mol/LKOH	180 °C, Hydrothermal treatment, 24 h	[55]		
	50 mL 22.5/25/30/35/40 M NaOH	280 °C, 15 h	[54]		
Molten salt etching	NH_4_HF_2_ molten salt	130 °C, 5 min	[57]	①Avoids the use of strong acids or bases, offering better chemical safety and environmental friendliness; ②Suitable for large-scale green production.	①Requires high-temperature operation, leading to higher energy consumption.
Other etching methods	NH_4_HF_2_ (6 g), CH_3_SO_3_H (6 mL) and NH_4_PF_6_ (6 g) dissolved in anhydrous DMSO (34 mL)	100 °C, 4 h	[61]	/	/
	40mL TMAOH solution	40 °C, Oil bath, 1 week	[70]		
	400 mg TFSI + 50 mL Acetic acid solution	150 W, Ultrasonic homogenizer stirring, 2 h	[71]		

**Table 2:** Midpoint environmental impacts for producing 1 g of Ti_3_C_2_T_x_ [93].

Impact Category	Unit	Path A [25,94]	Path B [95]	Path C [96]	Path D [97]	Path E [98]	Path F [99]	Path G [100]
Global warming	kg CO_2_ eq	1.44 × 10^00^	4.91 × 10^00^	7.08 × 10^00^	8.42 × 10^00^	7.54 × 10^00^	1.58 × 10^01^	1.40 × 10^01^
Stratospheric ozone depletion	kg CFC11 eq	5.05 × 10^−07^	1.61 × 10^−06^	2.57 × 10^−06^	3.00 × 10^−06^	2.82 × 10^−06^	5.44 × 10^−06^	5.33 × 10^−06^
Ionizing radiation	kBq Co-60 eq	2.27 × 10^−01^	7.52 × 10^−01^	1.50 × 10^00^	1.77 × 10^00^	1.82 × 10^00^	1.96 × 10^00^	3.27 × 10^00^
Ozone formation, human health	kg NO_x_ eq	2.11 × 10^−03^	7.95 × 10^−03^	9.98 × 10^−03^	1.24 × 10^−02^	9.72 × 10^−03^	2.28 × 10^−02^	1.94 × 10^−02^
Fine particulate matter formation	kg PM2.5 eq	2.88 × 10^−03^	1.04 × 10^−02^	1.63 × 10^−02^	1.96 × 10^−02^	1.76 × 10^−02^	2.79 × 10^−02^	3.30 × 10^−02^
Ozone formation, terrestrial ecosystems	kg NO_x_ eq	2.16 × 10^−03^	8.13 × 10^−03^	1.02 × 10^−02^	1.26 × 10^−02^	9.94 × 10^−03^	2.32 × 10^−02^	2.00 × 10^−02^
Terrestrial acidification	kg SO_2_ eq	3.99 × 10^−03^	1.57 × 10^−02^	2.04 × 10^−02^	2.48 × 10^−02^	1.99 × 10^−02^	4.18 × 10^−02^	3.80 × 10^−02^
Freshwater eutrophication	kg P eq	8.01 × 10^−04^	2.83 × 10^−03^	4.65 × 10^−03^	5.53 × 10^−03^	5.21 × 10^−03^	8.24 × 10^−03^	9.75 × 10^−03^
Marine eutrophication	kg N eq	6.09 × 10^−05^	2.49 × 10^−04^	3.41 × 10^−04^	4.07 × 10^−04^	4.25 × 10^−04^	9.86 × 10^−04^	7.59 × 10^−04^
Terrestrial ecotoxicity	kg 1,4-DCB	4.05 × 10^00^	1.72 × 10^01^	2.76 × 10^01^	2.54 × 10^01^	1.86 × 10^01^	4.29 × 10^01^	4.38 × 10^01^
Freshwater ecotoxicity	kg 1,4-DCB	1.05 × 10^−01^	3.50 × 10^−01^	5.75 × 10^−01^	6.39 × 10^−01^	5.50 × 10^−01^	1.24 × 10^00^	1.05 × 10^00^
Marine ecotoxicity	kg 1,4-DCB	1.34 × 10^−01^	4.51 × 10^−01^	7.35 × 10^−01^	8.16 × 10^−01^	6.99 × 10^−01^	1.59 × 10^00^	1.34 × 10^00^
Human carcinogenic toxicity	kg 1,4-DCB	1.61 × 10^−01^	7.90 × 10^−01^	8.17 × 10^−01^	1.09 × 10^00^	7.09 × 10^−01^	1.73 × 10^00^	1.51 × 10^00^
Human noncarcinogenic toxicity	kg 1,4-DCB	1.50 × 10^00^	5.46 × 10^00^	8.71 × 10^00^	9.69 × 10^00^	8.31 × 10^00^	1.75 × 10^01^	1.65 × 10^01^
Land use	M^2^a crop eq	2.88 × 10^−02^	9.82 × 10^−02^	1.56 × 10^−01^	1.84 × 10^−01^	1.71 × 10^−01^	2.80 × 10^−01^	3.28 × 10^−01^
Mineral resource scarcity	kg Cu eq	8.39 × 10^−03^	3.40 × 10^−02^	3.49 × 10^−02^	4.16 × 10^−02^	3.09 × 10^−02^	1.23 × 10^−01^	5.89 × 10^−02^
Fossil resource scarcity	kg oil eq	3.42 × 10^−01^	1.17 × 10^00^	1.77 × 10^00^	2.15 × 10^00^	1.92 × 10^00^	3.08 × 10^00^	3.61 × 10^00^
Water consumption	m^3^	1.23 × 10^−02^	3.62 × 10^−02^	5.10 × 10^−02^	5.93 × 10^−02^	4.95 × 10^−02^	1.34 × 10^−01^	2.53 × 10^−01^
